# Cancer stem cells: Bridging microenvironmental interactions and clinical therapy

**DOI:** 10.1002/ctm2.70406

**Published:** 2025-07-15

**Authors:** Huiling Wang, Junshu Li, Fei Du, Hongxin Deng

**Affiliations:** ^1^ Department of Biotherapy, Cancer Center and State Key Laboratory of Biotherapy, West China Hospital Sichuan University Chengdu Sichuan People's Republic of China

**Keywords:** cancer stem cells, immune escape, therapeutic resistance, tumour microenvironment

## Abstract

Cancer stem cells (CSCs) are a core subpopulation of tumour tissues exhibiting stem cell properties. Although they constitute only a minority of tumour cells, CSCs have become a central force driving tumourigenesis, metastasis, recurrence and resistance to therapy, owing to their abilities for self‐renewal, multi‐lineage differentiation and tumour‐initiating ability. Recent advances in multi‐omics analysis, lineage tracing and single‐cell sequencing technologies have systematically elucidated the dynamic biology of CSCs, including their epigenetic plasticity, metabolic adaptations and phenotypic heterogeneity, which depend on their ecological niche. In this review, we summarise the biological properties of CSCs, the molecular regulatory mechanisms and the complex interactions with the tumour microenvironment. We focus on strategies to target CSCs and the clinical translational challenges associated with these approaches. Collectively, this review organically integrates basic mechanisms and clinical translational research on CSCs, offering a comprehensive framework for understanding tumour biology and developing precision therapeutic strategies.

**Highlights:**

- Systems integration of CSC biology: Elucidate the dynamic properties, self‐renewal, plasticity and drug resistance.
- Microenvironmental interactions: Bidirectional interactions between CSCs and other cells, providing insights into niche‐driven immune evasion and metastasis.
- Therapeutic strategies: Evaluate emerging therapies targeting CSC‐specific markers and signals.
- Future directions: Challenges are discussed, with proposed solutions including multi‐omics‐guided precision medicine and microenvironment remodelling.

## Graphical abstract

Cancer stem cells (CSCs) drive tumor progression through their dynamic self‐renewal capacity, epigenetic plasticity, and drug resistance. These cells interact bi‐directionally with the tumor ecosystem to promote immunosuppressive reprogramming and metastatic spread. We systematically evaluated current targeting of CSC‐specific markers, signaling pathways and tumor microenvironment interactions, and clinical therapeutic strategies.

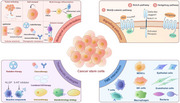

## 1 INTRODUCTION

Cancer poses a major challenge to modern medicine, with its complexity rooted in genetic heterogeneity, adaptive drug resistance mechanisms and dynamic interactions within the tumour microenvironment (TME).^1^ Although there have been advancements in conventional therapies, including surgery, chemotherapy, radiotherapy and targeted treatments, the recurrence of tumours and metastasis persists as the primary causes of mortality.^2^ The key driver of these recalcitrant behaviours lies in a unique subpopulation of cells referred to as cancer stem cells (CSCs), which are also known as tumour stem cells (TSCs) or tumour‐initiating cells (TICs). The concept was first conceptualised in the 19th century and validated in leukaemia by Bonnet and Dick.^3^ These cells, although rare, exhibit stem cell‐like properties, such as self‐renewal, multi‐lineage differentiation and adaptive plasticity, allowing them to evade eradication and regenerate heterogeneous tumours.^4^ The ‘cancer stem cell hypothesis’ fundamentally challenges the conventional view of tumour homogeneity, suggesting that CSCs are able to maintain tumour heterogeneity by manipulating both genetic and non‐genetic factors to promote tumour growth and resistance to therapy.^5^, ^6^ Unlike a large number of differentiated cancer cells, CSCs have the intrinsic ability to resist apoptosis, detoxify chemotherapeutic agents via ALDH1 and ABC transporters and dynamically change their phenotype in response to microenvironmental cues.^7^ Recent breakthroughs in multi‐omics technologies, including single‐cell sequencing and spatial transcriptomics,^8^, ^9^ have revealed the molecular complexity of CSCs biology. These tools describe how epigenetic modifications, metabolic reprogramming and ecotope‐specific signalling pathways converge to maintain the stemness of CSCs. In addition, the plasticity of CSCs illustrates their reversible transition between epithelial and mesenchymal states, as well as between quiescent and proliferative phases, which complicates therapeutic targeting.^10^

The microenvironment of tumours includes various components such as stromal cells, immune infiltrates, elements of the extracellular matrix and microbiota, forming a symbiotic ecosystem within the tumour. The tumour environment serves as both a refuge and a guide for CSCs.^11^ Tumour‐associated macrophages (TAMs) that are polarised towards the M2 phenotype release immunosuppressive cytokines, including transforming growth factor‐beta (TGF‐β) and IL‐10, thereby shielding CSCs from immune surveillance.^12^ Pericytes and cancer‐associated fibroblasts (CAFs) contribute to ecotone formation by providing metabolites such as methionine or activating pro‐survival pathways such as PDGFR‐β/GPR91.^13^ Interestingly, the gut microbiota has emerged as an unexpected regulator of CSC dynamics. For example, in colorectal cancer, colistin‐producing *Escherichia coli* strains induce genomic instability and upregulate stemness markers such as CD133 and OCT4.^14^ These interactions highlight the TME's role as a sanctuary and signalling hub for CSC. Clinically, eradication of CSCs remains elusive. Conventional therapies typically enrich the CSCs population by eliminating their differentiated progeny, inadvertently selecting therapy‐resistant clones. In addition, compensatory crosstalk between signalling pathways limits the efficacy of single therapies. Emerging strategies targeting CSC aim to dismantle the CSC‐TME alliance through multiple pathways, such as combining immune checkpoint inhibitors with CSC‐specific vaccines,^15^ nanotechnology‐supported delivery of pathway inhibitors,^16^ and metabolic interventions targeting glycolysis or iron‐dependent cell death sensitivity.^17^ However, challenges remain, including targeted toxicity to CSCs, intra‐tumour heterogeneity and the lack of biomarkers to track the dynamics of CSCs in real‐time.

This review aims to provide a comprehensive overview of CSCs, covering multiple aspects, from basic research to clinical application. It is going to start with the definition and characteristics of CSCs. The review will then revisit the historical context of CSC research and summarise the latest advances in current scientific frontiers, including the molecular mechanisms of CSCs, their interactions with the TME and their roles in tumour initiation, progression and treatment. Additionally, this review will discuss the potential applications of CSCs in cancer therapy and explore emerging therapeutic strategies targeting CSCs. By synthesising existing literature and research findings, this review aims to explore the role of CSCs in cancer therapy from a comprehensive perspective and outline promising future research directions.

## 2 THE CONCEPT OF CSCS AND THEIR CHARACTERISTICS

### 2.1 Concept formation of CSCs

The concept of CSCs originated in the late 19th century, when scientists first observed that some tumour cells displayed properties similar to those of stem cells. However, it was not until the 1990s that this concept gained extensive attention and research.^18^ In 1997, Dick et al. identified and isolated CSCs from acute myeloid leukaemia (AML) patients for the first time, demonstrating that these cells could reinitiate the same disease in mice.^3^ This breakthrough challenged the notion that all cancer cells had equal potential for tumour progression. In recent years, advancements in technologies such as single‐cell sequencing and organoid modelling have greatly advanced our understanding of the biology of CSCs.^19^, ^20^ These innovations have drastically improved our comprehension of the biology underlying CSCs. The integration of these cutting‐edge methods has enabled researchers to delve deeper into the unique properties and behaviours of CSCs, thus expanding our knowledge of their role in tumourigenesis and potentially informing more effective therapeutic strategies. Consequently, the study of CSCs has emerged as a crucial area of investigation within the larger context of cancer biology, providing valuable insights that could lead to improved treatments for various malignancies.

CSCs possess dual functional capacities: they maintain self‐renewal through asymmetric division while simultaneously differentiating into heterogeneous tumour cell populations, thereby establishing the hierarchical architecture of tumours.^21^ Furthermore, CSCs exhibit dynamic plasticity, enabling them to adapt phenotypically in response to microenvironmental stresses. Through intricate crosstalk with stromal components and immune cells, CSCs actively construct specialised survival niches.^22^

**FIGURE 1:**
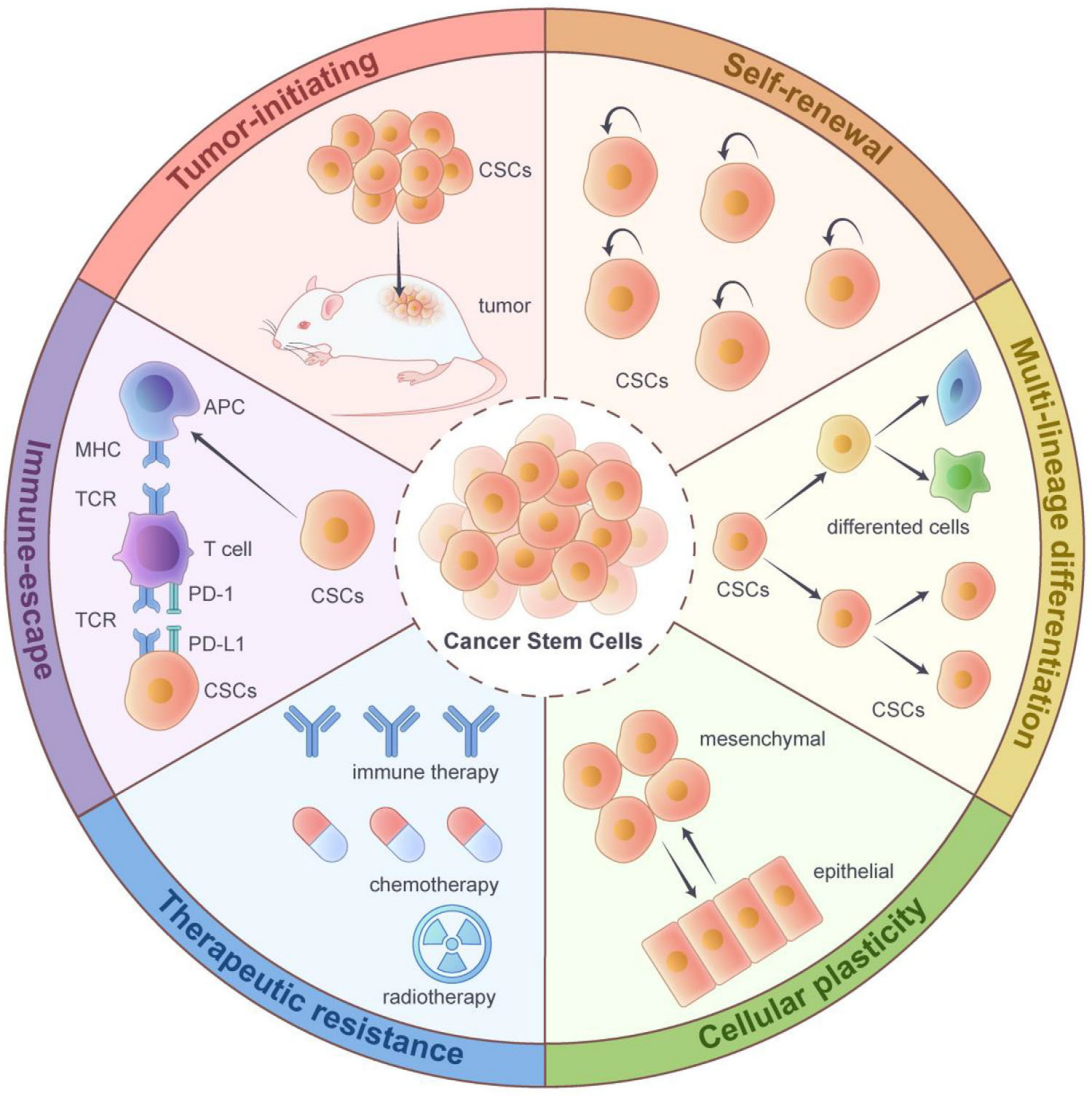
The core biological characterisation of cancer stem cells (CSCs). CSCs differ from differentiated tumour cells in several key features, including tumour initiation ability, self‐renewal, multi‐lineage differentiation, cell plasticity, therapeutic resistance and immune escape. CSCs have the ability to initiate and promote tumour formation when transplanted into suitable hosts. In addition, CSCs have the ability to produce offspring cells that are identical to the parent cells during division, while maintaining their undifferentiated state and tumourigenic potential. The characteristic of multi‐lineage differentiation leads to the production of heterogeneous cell populations in CSCs, and cellular plasticity enables them to dynamically transition between different functional states (quiescent/activated) or phenotypes (epithelial/mesenchymal). Moreover, ABC transporters (ABCG2, ABCG5), autophagy (ATG5/BECN1) and ALDH1‐driven detoxification and metabolic adaptation (such as enhanced glycolysis) mediate therapeutic resistance. Additionally, programmed death‐ligand 1 (PD‐L1) expression, secretion of immunosuppressive cytokines (interleukin‐10 [IL‐10], transforming growth factor‐beta [TGF‐β]), low major histocompatibility complex class I (MHC‐I) levels, recruitment of regulatory T cells (Tregs) and M2 macrophages induce immune escape.

Collectively, the core functional attributes of CSCs‐continuous self‐renewal and multidirectional differentiation potential are the fundamental basis of their tumour‐initiating ability. Their dynamic plasticity and ecological niche dependence, in turn, provide key adaptive mechanisms to ensure that they maintain this capacity in adverse environments, including therapeutic stress. Together, these interrelated features constitute an integrative mechanism for understanding the core biological issues of tumour heterogeneity, therapeutic resistance and recurrence and metastasis (Figure 1).

### 2.2 Characteristics of CSCs: Tumour‐initiating ability

Tumour initiation refers to the ability of specific tumour cells to initiate and contribute to tumour formation when transplanted into a suitable host environment. When the role of CSCs was first identified in the 1990s, tumour initiation was defined as the initial hallmark of CSCs and became a primary focus of CSCs research. For example, in AML, only a subpopulation of CD34^+^/CD38^−^ CSCs could reconstitute the leukaemia model in immunodeficient mice, whereas differentiated CD34^−^ cells lacked tumourigenic potential.^3^ These findings in haematologic malignancies have spurred extensive research on CSCs, leading to the discovery that ‘tumour‐initiating capacity’ is also significant in solid tumours. For instance, transplantation into CD44^+^/CD24^−^/ALDH1^+^ CSCs from breast cancer into mouse mammary fat pads resulted in the formation of foci that closely resembled the histological features of the primary tumour. Notably, only 1/1000 of the number of CSCs was required compared to non‐CSCs to achieve this effect.

The expression of specific genes is strongly correlated with tumour‐initiating capacity. For instance, the protein adenosine deaminase RNA‐specific binding protein (ADAR1) promotes ganglioside catabolism and drives glioblastoma initiation by enhancing ganglioside GM2 activator (GM2A) expression.^23^ Furthermore, ADAR1 induces an arginine to glycine (R701G) mutation in hepatic CSCs through its regulation of RNA editing on the glioma‐associated oncogene homolog 1 (GLI1), which improves the nuclear localisation of GLI1 and promotes tumour initiation.^24^ The transcription factor SOX9 plays a crucial role in regulating mammary stem and progenitor cells. Cui et al. found that in an in vivo limiting dilution inhibition assay, tumours could only be generated by C3/TAg tumour cells that express SOX9, with SOX9^high^ cells exhibiting a fourfold increase in tumour‐inducing capacity compared to their SOX9^low^ counterparts.^25^ Furthermore, Ravindran Menon et al. demonstrated that in melanoma, colon and pancreatic cancers, blocking CDK1 expression reduced the phosphorylation, localisation and transcriptional activity of the pluripotency‐associated transcription factor SOX2, thereby inhibiting tumour initiation.^26^ In non–small‐cell lung cancer (NSCLC), the expression level of the *Fat1* gene is associated with tumour‐initiating capacity. *Fat1* activates the Hippo signalling pathway by promoting nuclear translocation of YAP1, which reduces the sphere‐forming ability and expression of tumour initiation markers in NSCLC cells, suggesting that *Fat1* can inhibit NSCLC tumour initiation.^27^

### 2.3 Characteristics of CSCs: Self‐renewal and multi‐lineage differentiation

Two other defining characteristics of CSCs are self‐renewal and multi‐lineage differentiation capacity. If tumour‐initiating capacity is the functional source of the definition of CSCs, then self‐renewal and multi‐lineage differentiation together form the biological basis of CSC tumour‐initiating capacity, and these processes are interdependent. Self‐renewal refers to their ability to generate progeny cells identical to their parental cells during division while retaining their undifferentiated state and tumourigenic potential. This characteristic is a central mechanism of CSCs‐driven tumour growth. Multi‐lineage differentiation refers to the ability of CSCs to differentiate into multiple tumour cell types under specific influences, such as endogenous mutations or external pressures, forming a heterogeneous tumour tissue hierarchy.

Four main models explain the mechanism of self‐renewal and multi‐lineage differentiation of CSCs. The Hierarchical organisation model is one of the initial frameworks for CSC research and is also known as the classical model.^28^ This model assumes that structured tissues exist within the tumour and that CSCs are located at the top, acting similarly to adult stem cells in tissue differentiation. These cells undergo asymmetric divisions, resulting in a daughter cell that retains stem cell properties and another that differentiates into other cell types. In a hierarchical tissue model, CSCs are less differentiated compared to more differentiated cancer cells. In this model, self‐renewal and multi‐lineage differentiation potentials are among the inherent properties of CSCs that drive long‐term tumour growth. As the researchers found, the expression of specific stem cell markers, such as CD133 and ALDH1, was restored in tumours following transplantation experiments.^29^

Another model is the clonal evolution model. This theory suggests that tumours evolve through mutation and natural selection in the host microenvironment (niche).^30^, ^31^ In this model, CSCs are considered to be clonal populations with specific mutations that give them characteristics such as self‐renewal. The model focuses on tumours being genetically heterogeneous and adaptive, with self‐renewal roles and multi‐lineage differentiation potential acting as competitive advantages conferred by mutations in response to microenvironmental signals, which in turn affects the outcome of stem cell divisions, generating 0, 1 or 2 new stem cells. Under physiological conditions, this model has been described in detail in the colorectal crypt,^32^, ^33^ stomach,^34^ and epidermis.^35^ During tumour progression, CSCs can become progressively independent of host microenvironmental signals through genetic mutations. This leads to enhanced self‐renewal, inhibition of differentiation and formation of superficial secondary structures dominated by CSCs. In hepatocellular carcinoma, methyltransferase 16 (METTL16) expression enhances the regulation of eIF3a transcription and promotes tumour proliferation.^36^ Similarly, in leukaemia, METTL16 enhances the stemness of leukaemic stem cells (LSCs) by mediating the m6A transcriptome and metabolic reprogramming.^37^ Furthermore, in lung cancer, METTL16‐mediated SLC7A11 in stem‐like cells is upregulated and activated by the stem cell transcription factor SOX2. This upregulation and activation contribute to the maintenance and function of lung CSCs.^38^

Additionally, the plasticity model considers CSCs as a transient state regulated by epigenetic mechanisms or microenvironmental factors such as hypoxia, inflammation or therapeutic stress.^15^, ^39^, ^40^, ^41^ In this model, self‐renewal and multi‐lineage differentiation are treated as temporary stress‐induced capabilities to enhance the adaptability of CSCs. For example, Chan et al. discovered that activation of Jak/STAT and FGFR inflammatory signalling can drive lineage plasticity in prostate cancer, such as the transformation of adenocarcinoma into neuroendocrine carcinoma, which originates from an epithelial population defined by a mixed luminal–basal phenotype.^42^ In malignant cutaneous squamous cell carcinoma (SCC), the nuclear receptor NR2F2 enhances tumour cell proliferation, epithelial–mesenchymal transition (EMT) and invasion through upregulated expression under microenvironmental stress.^43^ Similarly, in epigenetic regulation of CSCs, the lncRNA HNF1A activated by c‐Myc transcription is highly expressed in gastric CSCs, thereby regulating β‐catenin expression.^44^ A newly identified lncRNA CASCADES acts as a super‐enhancer of SOX2 and participates in the epigenetic regulation of glioma stem cells.^45^ In addition, the mitochondria‐encoded circular RNA (circRNA) can recruit the Tat‐interacting protein 60 complex, thus promoting the expression of the *MAFF* gene.^46^

Recently, Song et al. proposed a new model for CSCs, the mimicry model, which is an adaptive strategy for cancer cells to mimic the stem cell state under stress by mimicking immune cells, vascular cells or viruses to evade immune cells and resist treatment.^47^ In this model, self‐renewal and multi‐lineage differentiation as adaptive strategies are the foundation for evolutionary deception for active maintenance (foundation for evolutionary deception). Isolated CSCs in cancer can evade immune surveillance by overexpressing programmed death‐ligand 1 (PD‐L1). Still, it has been found that some subpopulations of cancer cells produce both programmed cell death‐1 (PD‐1) and PD‐L1, such as hepatocellular carcinoma cells expressing PD‐1, which directly maintain an anti‐tumour immune response without the need for immune cell involvement.^48^, ^49^ In addition, glioma stem cells also differentiate into endothelial cells under hypoxic conditions, forming vascular networks that promote tumour blood supply and metastasis.^50^, ^51^

### 2.4 Characteristics of CSCs: Cell plasticity

Cellular plasticity, a fundamental process in embryonic development, denotes the capability of cells to adopt various phenotypes in reaction to external stimuli or environmental changes, all while not experiencing any genetic modifications.^52^ During cancer progression, tumour cells can switch between cellular states, primarily through the function of cellular plasticity. This process largely promotes intra‐tumour heterogeneity, increases the adaptability of tumour cells to their microenvironment and greatly contributes to tumour growth, metastasis and therapeutic resistance.

Traditionally, CSCs were viewed as a scarce group of cells exhibiting restricted plasticity. Nonetheless, increasing experimental evidence suggests that more differentiated cancer cells have the ability to transition back to a less differentiated state when exposed to specific conditions or stimuli. This suggests that CSCs represent a cellular state rather than a fixed condition.^4^, ^53^ The plasticity of CSCs is manifested by their dynamic transitions between different functional states (quiescent/activated) or phenotypes (epithelial/mesenchymal), which is a central strategy for tumours to adapt to microenvironmental stresses such as chemotherapy, hypoxia and immune attack.

This concept was confirmed in a breast cancer study where researchers isolated cell populations exhibiting stem cell‐like, basal‐like or luminal‐like phenotypes from breast cancer cell lines. In vitro experiments showed that all three subpopulations could generate cells of the other two phenotypes, gradually converging cell type proportions in the cultures to those observed in the original breast cancer cell lines.^54^ This phenotype switching is random and not determined by the initial cell phenotype. Importantly, while only stem cell‐like cells efficiently generate tumours under standard conditions, all three phenotypes become tumourigenic when the environment is altered (e.g., after irradiation and injection). This suggests that the state of CSCs and non‐CSCs is not fixed but rather an adaptation to external environmental cues.

Researchers have observed that more differentiated cancer cells can redifferentiate into Lgr5^+^ cells, thereby replenishing the stem cell pool in colorectal cancer. This dedifferentiation process allows tumours to repair and regenerate themselves when stem cells are disturbed.^20^, ^55^ However, glioblastomas appear to follow a more unidirectional developmental trajectory. In a glioblastoma mouse model, the removal of CSCs suppressed tumour development and extended survival, while no regeneration of new CSCs from other cell types was noted.^56^ The reprogramming of differentiated glioblastoma cells into CSCs was only possible when four essential transcription factors‐POU3F2, SOX2, SALL2 and OLIG2 were re‐expressed.^57^ These findings suggest that glioblastoma development is highly directional and irreversible.

### 2.5 Characteristics of CSCs: Therapeutic resistance and immune escape

The therapeutic resistance and immune escape properties of CSCs are central to their biological behaviours, which sustain malignant tumour progression. Both of these properties are key functional characteristics of CSCs that interact with the TME through complex molecular mechanisms to drive tumour recurrence and metastasis jointly.

In terms of therapeutic resistance, CSCs are resistant to chemotherapy, radiotherapy and targeted therapies through multiple strategies, including the dynamic regulation of metabolism, epigenetic and microenvironmental interactions. For example, the quiescent state (G0 phase) of CSCs renders them insensitive to chemotherapeutic agents that act on proliferating cells, such as the quiescent LGR5^+^p27^+^ CSCs in colorectal cancer, which are resistant to chemotherapy and drive recurrence.^58^ In addition, CSCs reduce intracellular drug concentrations through high expression of drug‐efflux pumps such as ABCG2 and ABCB5 proteins. CD133^+^ lung cancer CSCs mediate vemurafenib resistance through ABCG2.^59^ Metabolic reprogramming of CSCs enhances their drug resistance, and NSCLC stem cells rely on methionine recycling to maintain histone methylation (H3K4me3/H3K27me3) to activate SOX2. Deprivation of methionine or inhibition of the key enzyme MAT2A suppresses their therapeutic resistance.^60^ CSCs enhance their drug resistance through epigenetic reprogramming. For example, glioblastoma stem cells express the methylase LSD1, which dynamically shuts down MYC, SOX2 and other stemness genes, allowing the cells to enter a ‘dormant state’ during drug treatment and reactivate the tumourigenic program after drug discontinuation.^61^

In terms of immune escape, CSCs evade immune surveillance by expressing immune checkpoint molecules, downregulating antigen presentation and constructing an immunosuppressive microenvironment. For example, lung cancer CSCs with high expression of SIRPγ remodel the immunosuppressive microenvironment and transmit immune escape signals by maintaining CD47 expression. Targeting SIRPγ therapy suppresses the CSLC phenotype and triggers phagocytosis of tumour growth in vivo.^62^ Downregulation of the differentiation factor LCOR in triple‐negative breast cancer (TNBC) CSCs results in antigen processing (APM) defects.^63^ SCC CSCs expressing CD80 can directly inhibit cytotoxic T cell activity by binding CTLA4. Following CTLA4 or TGF‐β blockade immunotherapy or CD80 ablation, CSCs become vulnerable and reduce tumour recurrence after ACT treatment.^64^ In addition, CSCs induce infiltration of myeloid‐derived suppression of cells (MDSCs), such as prostate CSCs, recruiting MDSCs by secreting CXCL5 via YAP signalling, creating an immune desert microenvironment.^65^

Due to the therapeutic resistance and immune escape properties of CSCs, tumour eradication requires targeting their ecological niche, and only by simultaneously destroying the triple barriers of metabolic dependence, epigenetic regulation and the immune microenvironment can we achieve durable clearance. For example, ALDH1A1‐responsive metabolic glycan labelling of CSCs in conjunction with click chemistry,^66^ or targeting the surface antigens of CSCs, CD44 and CD133, by CAR‐T cells.^67^, ^68^, ^69^

## 3 BIOMARKERS OF CSCS

The precise identification of CSCs markers holds profound significance for the clinical management of cancer. In recent years, substantial advancements have been achieved in CSC marker research. A diverse array of markers has been unearthed, each playing an integral role in maintaining the biological properties of CSCs, modulating the TME and influencing the intricate processes of tumourigenesis and tumour progression. Based on their functional attributes and characteristics, these markers can be broadly categorised into several groups: surface markers, transcription factors, intracellular functional markers and immune‐related markers (Table 1). We provided a detailed and in‐depth exploration of these CSC marker types in this section.

**TABLE 1:** Classification and function of cancer stem cell biomarkers.

Category	Biomarker	Cancer type	Function	References
Surface marker	CD44	Breast cancer	Activates Ras‐MAPK, P13K‐Akt, STAT3 signalling pathways; express SOX2 and OCT4	^70^, ^71^, ^72^
		Colorectal cancer	Enhances of metastasis by activation of the β‐catenin signalling pathway, interact with stromal cells to promote angiogenesis	^73^
		Lung cancer	Regulates GPR124 expression to promote endothelial cell migration and induce brain metastasis	^74^
		Prostate cancer	Regulates the expression of miR‐9‐5p	^75^
		Oral cancer	Activates of NRF2 signalling to enhance reactive oxygen species	^76^
	CD133	Breast cancer	Regulates of Wnt and PI3K‐Akt signalling pathways	^77^
		Lung cancer	Regulates vemurafenib resistance through ABCG2; Activates PI3K/Akt/mTOR signalling	^59^, ^78^
		Glioblastoma	Activate of JNK‐STAT3 signalling and induction of immunosuppressive by TFPI2	^79^
		Liver cancer	Labelled CSC‐like proliferating cells, clonally expanded and expressing EMT markers in tumour; activates TACE/ADAM17‐dependent Notch signalling pathway	^80^, ^81^
	CD34	Acute myeloid leukaemia	Proliferates extensively and produce heterogeneity in mice	^3^, ^82^
		Chronic myeloid leukaemia	Associated with tyrosine kinase inhibitor resistance	^83^
	EpCAM	Colorectal cancer	EpCAM‐cells with mesenchymal features and high metastatic potential	^84^
		Liver cancer	Activate the Wnt/β‐catenin signalling and serves as a poor prognostic marker along with AFP; Blocks the expression of CEACAM1 can increase the toxicity of NK cells	^85^, ^86^
		Gastric cancer	CD24^+^CD44^+^CD54^+^EpCAM^+^ cells have higher tumourigenicity and metastatic capacity;	^87^, ^88^
Transcription factor	SOX2	Glioblastoma	Reprogramming of differentiated glioblastoma	^89^
		Colorectal cancer	Activates β‐catenin signalling and Beclin1 to promotes colorectal cancer stemness	^90^
		thyroid cancer	Regulated self‐renewal and maintenance of stem cell properties along with BMI‐1	^91^
		Cervix cancer	Synergises with OCT4 to maintain tumourigenicity of CSCs	^92^
	NANOG	Pancreatic cancer	Regulated with KLF4 and SOX2 to promote stemness and drives gemcitabine resistance	^93^
		Lung cancer	Activates the P13K/Akt/mTOR signalling pathway and co‐localises with CD133 and CD44	^78^
		Pan‐cancer	Prevents the proteasomal degradation of HDAC1	^94^
	OCT4	Ovarian cancer	Regulates notch signalling pathway and resistants to platinum‐based chemotherapy	^95^
		Glioblastoma	Regulates CD133 and nestin expression, inhibits GFAP5 expression	^96^
	BMI‐1	Thyroid cancer	Promotes tumour cell self‐renewal with SOX2 via the Hh signalling pathway	^91^
		Breast cancer	Inhibits P16INK4α and P14ARF expression	^97^
		Liver cancer	Promotes radioresistance in hepatocellular carcinoma patients	^98^
		Larynx cancer	Reduced differentiation of CSCs and enhanced chemotherapeutic effects of paclitaxel	^99^
Intracellular functional marker	ALDH1	Breast cancer	Decreases intracellular pH and promotes TAK1 phosphorylation to activate NF‐κB	^100^, ^101^
		Colorectal cancer	Enhances oxidative stress tolerance and lipid metabolism	^102^, ^103^
		Lung cancer	Activates the MEK/ERK signalling pathway and increases DR4 and DR5 expression	^104^
	ABCB5	Melanoma	Blocks TMZ‐induced inhibition of G2/M phase block and increases cell death	^105^
		Glioblastoma	Maintenance of slow cellular cycling through the IL‐1β/IL8/CXCR1 signalling pathway	^70^, ^106^
	ABCG2	Pancreatic cancer	Mediates gemcitabine resistance	^107^
		Pan‐cancer	Mediates multidrug resistance through efflux of chemotherapeutic agents	^59^, ^108^
Immune‐related marker	PD‐L1	Lung cancer	Jak/STAT3/PDL‐L1 signalling pathway inhibits ATM reversal of EMT	^109^
		Ovarian cancer	Correlates with CD44 and LGR5 expression and is involved in tumour recurrence	^110^
		Breast cancer	Interacts with Frizzled 6 to activate β‐catenin and form a positive feedback loop	^111^
		Liver cancer	PD‐L1^+^ M2 macrophages expressing TGF‐β1	^48^, ^49^, ^107^

### 3.1 Surface markers

CD44 is a highly conserved transmembrane glycoprotein encoded by the *CD44* gene. This gene undergoes selective splicing to produce multiple isoforms, including standard (CD44s) and variant (CD44v) forms. Through its interaction with ligands—including hyaluronic acid (HA) and osteoblasts—the extracellular domain of CD44 activates downstream signalling pathways such as Ras‐MAPK, PI3K‐AKT and STAT3.^70^ These pathways regulate cell adhesion, proliferation, migration and drug resistance. The function of CD44 is highly context‐dependent and often synergises with other molecules, such as CD24 and CD133. Clark and colleagues were among the first to identify that CD44^+^CD24^−^/low breast cancer CSCs were capable of initiating tumours, whereas CD44^+^CD24^+^ cells were virtually incapable of forming tumours. CD44^+^CD24^−^/low cells account for 11%–35% of breast cancer cells, suggesting that they are a small but essential subpopulation of stem cells responsible for maintaining tumour heterogeneity and treatment resistance.^71^ Liu et al. also found that CD44^high^CD24^low^ breast CSCs maintain their stem cell status by enhancing the expression of *SOX2* and *OCT4* through TAZ‐NANOG phase separation.^72^ In colorectal cancer, CD44v6^+^ CSCs significantly enhance metastasis by activating the β‐catenin signalling pathway. Moreover, CD44^+^ cells interact with stromal cells to promote angiogenesis by secreting VEGF, thereby supporting tumour growth and distant colonisation.^73^

CD133, also known as Prominin‐1, is a conserved CSCs marker across cancer types. It maintains stem cell properties and drives malignant progression in various tumours through multiple mechanisms, including EMT, metabolic reprogramming, immune escape and microenvironmental remodelling. CD133 is highly expressed in breast CSCs and has been shown to regulate the Wnt and PI3K‐Akt signalling pathways, thereby promoting the self‐renewal and survival of CSCs. In addition, CD133 can be used as a marker for targeted therapies. For example, antibody–toxin couplers, such as CD133 antibody coupled to paclitaxel, significantly inhibited local tumour recurrence in a mouse model.^77^ It was found that in hepatocellular carcinoma, Prom1 marks proliferative tumour cells with characteristics of CSCs. Lineage tracing has demonstrated that these cells undergo clonal expansion in situ within primary tumours. Moreover, Prom1^+^ cells express EMT markers in tumours generated after transplantation, suggesting that these cells have the potential for both differentiation and transdifferentiation.^80^ CD133^+^ glioblastoma stem cells (GSCs) activate the JNK‐STAT3 pathway by secretion of tissue factor inhibitor 2 (TFPI2). This activation maintains stem cell properties and induces the infiltration of immunosuppressive microglial cells.^79^

CD34 is an extensively glycosylated transmembrane protein that was first recognised as a marker on the surface of haematopoietic stem cells (HSCs). In the field of oncology, CD34 serves as a classical CSC marker in leukaemia and is closely associated with disease onset, progression and drug resistance. In the 1990s, Bonnet and Dick identified a rare population of CD34^+^CD38^−^ CSCs in AML (.02%–2%). These cells were able to proliferate extensively in NOD/SCID mice, producing the heterogeneity observed in the originating tumour. These findings highlight the potential of normal stem cells to produce various lineages.^112^ Furthermore, in chronic myeloid leukaemia (CML), CD34^+^ cells are enriched in CML patients, and their presence correlates with tyrosine kinase inhibitor resistance.^83^ In solid tumours, CD34 expression is mainly associated with tumour angiogenesis, but its specificity as a CSC marker is low. For example, in glioblastoma^113^ and colorectal cancer,^114^ CD34^+^ cells are involved in tumour angiogenesis by differentiating into endothelial cells, thereby promoting tumour growth. The precise function of CD34 proteins in these solid tumour settings remains to be fully elucidated. Further studies are needed to investigate the role of CD34 in solid tumourigenesis, which may lead to new avenues for targeted therapy.

Epithelial cell adhesion molecule (EpCAM) is an epithelial phenotypic marker that identifies populations with stem cell properties in various solid tumours. Its absence (EpCAM^−^) is often associated with EMT, metastasis and treatment resistance. In colorectal cancer, EpCAM^+^ cells represent a typical epithelial phenotype, whereas EpCAM^−^ cells exhibit mesenchymal features and higher metastatic potential. It has been shown that after chemotherapy, EpCAM^−^ cells acquire a persistent dormant phenotype through activation of the foetal stem cell gene signature, which in turn promotes tumour recurrence.^84^ In hepatocellular carcinoma, EpCAM^+^ CSCs maintain self‐renewal capacity through activation of the Wnt/β‐catenin signalling pathway. Together with AFP, these cells act as markers of poor prognosis.^85^ A cohort study of 127 untreated gastric cancer patients demonstrated that CD24^+^CD44^+^CD54^+^EpCAM^+^ cells are bona fide gastric CSCs. This expanded phenotype is associated with higher tumourigenicity and a positive correlation with tumour metastasis, suggesting that these cells may serve as a prognostic marker for gastric cancer.^87^

### 3.2 Transcription factors

SOX2 acts as a core stemness transcription factor, helping to maintain the undifferentiated state of stem cells. In tumours, high expression of SOX2 is closely associated with tumour recurrence, treatment resistance and poor prognosis. As a core neurodevelopmental transcription factor, SOX2 expression reprograms differentiated glioblastoma cells to acquire GSC phenotype and function.^115^ In colorectal cancer, SOX2 promotes stemness, chemotherapy resistance and EMT in colorectal CSCs. This is achieved through activating the β‐catenin signalling pathway and the autophagy‐associated protein Beclin1, which enhances tumour invasion and metastasis.^90^ Additionally, SOX2 synergises with the transcription factor OCT4 to maintain the tumourigenic capacity of cervical CSCs, driving tumour proliferation and recurrence.^92^

NANOG serves as a critical transcription factor involved in both embryogenesis and tumourigenesis, exhibiting overexpression in the majority of CSCs.^116^ Within CSCs, NANOG promotes metastasis, self‐renewal, tumourigenesis, tumour recurrence and drug resistance. Elevated levels of NANOG are significantly linked to advanced disease stages, reduced overall survival rates and lower differentiation across multiple cancer types.^117^ In pancreatic cancer, NANOG forms a regulatory network with other transcription factors, such as KLF4 and SOX2, that promotes stem cell maintenance in CSCs and drives gemcitabine resistance through the upregulation of c‐Myc.^93^ NANOG promotes cell survival and cisplatin resistance in lung cancer by activating the PI3K/Akt/mTOR signalling pathway in lung CSCs. Its expression co‐localises with CSC markers CD133 and CD44.^78^ In addition, NANOG regulates histone deacetylase 1 (HDAC1) and influences transcriptional activity. NANOG phosphorylates and inactivates CHFR, an E3 ubiquitin ligase, through the AKT signalling pathway, which prevents the proteasomal degradation of HDAC1. This leads to the accumulation of HDAC1. Consistent with this, the accumulation of HDAC1 is related to metastasis in various cancers, and inhibition of AKT signalling results in the degradation of HDAC1.^94^

OCT4 is a core pluripotency transcription factor that maintains the self‐renewal capacity of CSCs. High expression of OCT4 is closely linked to tumour recurrence, poor prognosis and treatment resistance, making it a key molecule for CSC characterisation. In ovarian cancer, OCT4 maintains the stemness of ovarian CSCs and is associated with platinum‐based chemotherapy resistance through the regulation of the Notch signalling pathway. Its co‐localisation with CD133 promotes tumour recurrence.^95^ In glioblastoma, OCT4 drives the self‐renewal of GSCs by regulating the expression of CD133 and Nestin while inhibiting the expression of differentiation‐related genes such as *GFAP*.^96^

BMI‐1 belongs to the Polycomb group (PcG) family of proteins and is a key member in epigenetic regulation. As a core transcription factor in CSCs, BMI‐1 plays a critical role in various cancers by regulating self‐renewal, repressing differentiation‐related genes and promoting drug resistance. In thyroid cancer, BMI‐1 is involved in self‐renewal and the maintenance of stem cell properties in conjunction with SOX2. The Hedgehog signalling pathway promotes the self‐renewal capacity of CSCs in thyroid tumours by regulating the expression of BMI‐1 and SOX2.^91^ BMI‐1 has been identified as a key transcription factor in maintaining CSC stemness and drug resistance in breast cancer. It promotes the self‐renewal and chemoresistance of CSCs by inhibiting the expression of tumour suppressor genes such as p16INK4a and p14ARF.^97^

### 3.3 Intracellular functional markers

Aldehyde dehydrogenases (ALDHs) belong to a group of enzymes responsible for detoxification. The ALDH family comprises 19 isoforms that are found to be active across various mammalian tissues. Each isoform exhibits a distinct expression profile, with many of them being overrepresented in a subset of cancer cells that display characteristics akin to stem cells. These enzymes play crucial roles in processes such as cell proliferation, differentiation, detoxification, survival, as well as in the metabolism of lipids and amino acids and the synthesis of retinoic acid. They are commonly used for CSC sorting based on their enzymatic activity (ALDEFLUOR assay).^118^ Mechanistically, ALDH enzymes shield cancer cells by converting harmful aldehydes into more soluble and less reactive carboxylic acids.^119^ Among these, ALDH1, especially isoform ALDH1A1, is a key marker for CSCs in breast,^100^ prostate,^120^ colon,^121^ and lung cancer.^104^ For example, ALDH1A1, which is highly expressed in breast CSCs, activates nuclear factor‐κB signalling and increases GM‐CSF secretion by decreasing intracellular pH to promote TAK1 phosphorylation. This leads to MDSC expansion and immunosuppression. In addition, the ALDH1A1 inhibitor disulfiram and the chemotherapeutic agent gemcitabine synergistically inhibit breast tumour growth and tumourigenesis by removing ALDH^+^ TICs and activating T cell immunity.^101^ In colon CSCs, high expression of ALDH1 maintains stem cell properties through enhanced oxidative stress tolerance and lipid metabolism. Inhibitors targeting ALDH1, such as DEAB, induce apoptosis in CSCs.^102^ Furthermore, the ALDH1A1‐responsive glycan precursor AAMCHO has been used to label colon CSCs for precision‐targeted therapy metabolically.^103^ In lung cancer, ALDH1 can increase the expression of death receptors 4 or death receptors 5 in ALDH1^+^ NSCLC cells by activating the MEK/ERK signalling pathway.^122^

ABC family transporter proteins are a superfamily of proteins that rely on ATP hydrolysis for energy to transport substances across membranes. In tumours, these proteins drive chemoresistance and relapse in various cancers by mediating drug efflux and maintaining the stemness of CSCs. Among them, ABCG2 has been extensively studied and was initially identified in multidrug‐resistant breast cancer cell lines as Breast Cancer Resistance Protein (BCRP).^123^ ABCG2 has been identified as a biomarker for CSCs in lung, pancreatic, hepatocellular, breast and ovarian cancers.^59^ Its mediation of multidrug resistance (MDR) through the efflux of chemotherapeutic agents, metabolites or toxins is one of the key factors in tumour treatment failure.^108^ Another family member, ABCB5, controls IL‐1β secretion in melanoma‐initiating cells through the IL‐1β/IL‐8/CXCR1 cytokine signalling circuit to maintain slow cycling in drug‐resistant cells.^105^ Besides, ABCB5 has also been identified as a marker of glioblastoma multiforme resistance by blocking the inhibition of TMZ‐induced G2/M‐phase block and increasing TMZ‐mediated cell death.^106^

### 3.4 Immune‐related markers

As a central driver of tumourigenesis, metastasis and recurrence, the immune escape ability of CSCs is a key mechanism of therapeutic resistance. CSCs actively shape an immunosuppressive microenvironment by expressing specific immunomodulatory molecules, evading immune surveillance and sustaining stemness. They are known to evade immune surveillance and maintain stemness by high expression of inhibitory receptors, such as PD‐L1, the secretion of non‐inflammatory cytokines, including IL‐4, IL‐10 and IL‐13, as well as TGF‐β, low expression of major histocompatibility complex class I (MHC‐I) and the prevention of immune cell infiltration into the TME. These mechanisms suppress the activity of effector T cells, antigen‐presenting cells and natural killer (NK) cells.^124^ In addition, low expression of MHC I allows CSCs to evade recognition by the immune system and avoid attack by CD8^+^ T cells. CSCs are also highly expressive of ligands for NK cell activation receptors such as MICA/B and ULPB, and NK therapies could target these ligands to explore the direct killing of CSCs.^125^

Current research efforts in marker studies of CSCs emphasise multidimensional targeting strategies. Integration of various marker‐related studies provides a solid theoretical basis for the development of targeted therapies. However, as shown by the studies covered in this review, the expression profiles and functional significance of many markers are highly tumour‐type specific. In particular, there are significant differences in CSC markers between solid tumours and haematological malignancies. For example, CD133 is often used as an important surface marker in solid tumours such as breast cancer and hepatocellular carcinoma^81^, ^126^ for identifying cell subpopulations with high tumourigenicity, but its function in AML remains to be further validated. Intracellular functional markers such as ALDH are widely used to enrich CSCs in breast cancer^118^ and colorectal cancer,^127^ but in AML, ALDH activity is only expressed in a few stem cell subpopulations. In contrast, surface markers such as CD34 are relatively more specific for LSCs in AML.^82^ Therefore, the application of CSC markers needs to be closely integrated with tumour types and molecular characteristics. Furthermore, the combination of universal and tumour‐specific markers across different tumour types should be further explored in the future to enhance the accuracy of diagnosis, treatment and prognosis.

## 4 SIGNALLING PATHWAYS IN REGULATING CSCS

**FIGURE 2:**
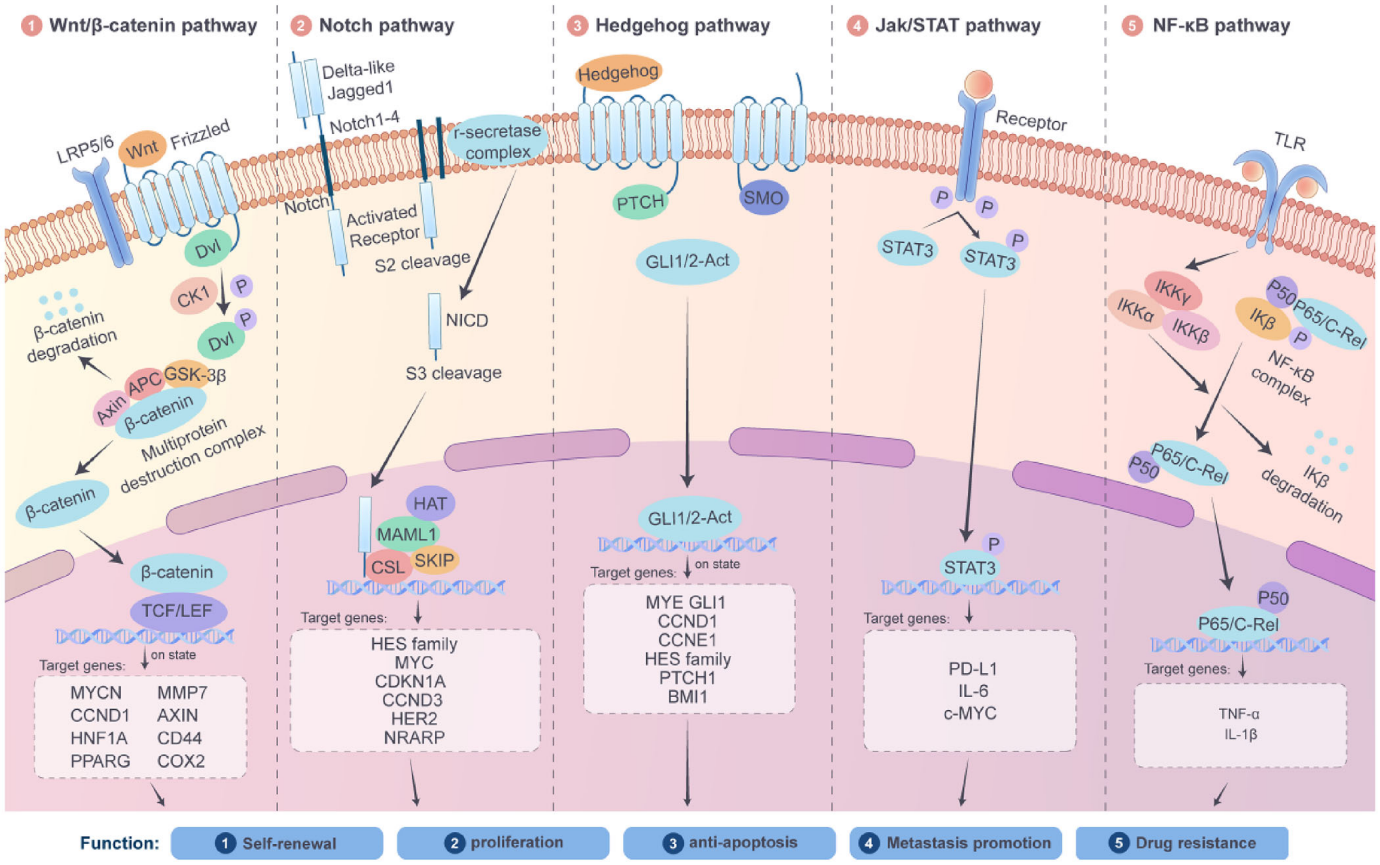
Regulatory network of key signalling pathways in cancer stem cells (CSCs). Control of CSCs maintenance is mainly through the following signalling pathways: 1. In the Wnt/β‐catenin pathway, Wnt protein binds to its receptors Frizzled and LRP to induce activation of Dishevelled, which mediates downstream gene activation via β‐catenin/T‐cell factor (TCF)/lymphoid enhancer‐binding factor (LEF), leading to activation of gene expression for cytoskeletal remodelling, regulation of pluripotency and cell differentiation; 2. The Notch pathway regulates differentiation and immune evasion through Notch intracellular domain (NICD)‐dependent activation of stemness genes (e.g., Hes1); 3. The Hedgehog (Hh) pathway drives metabolic reprogramming and drug efflux via Gli‐mediated upregulation of ABCG2; 4. Jak/STAT3 pathways facilitate immune escape by inducing PD‐L1 and immunosuppressive cytokines; 5. The NF‐κB pathway mainly mediates inflammatory responses, such as those in the tumour necrosis factor‐α (TNF‐α) and interleukin‐1β (IL‐1β) signalling pathways, creating a favourable microenvironment for CSCs survival.

The maintenance of CSC pluripotency greatly depends on a series of evolutionarily conserved signalling pathways, including the Wnt/β‐catenin, Notch, Hedgehog (Hh), STAT3 and NF‐κB pathways (Figure 2). Through their intricate and sophisticated molecular mechanisms, these pathways precisely regulate the proliferation, metabolic adaptation and microenvironment interactions of CSCs. As a result, they have become crucial areas of research in cancer‐targeted therapy.

### 4.1 The Wnt/β‐catenin pathway: A core pathway for pluripotency maintenance and drug resistance

The Wnt/β‐catenin pathway is pivotal in embryonic development and maintaining adult stem cell homeostasis.^128^ In CSCs, aberrant activation of this pathway can endow them with self‐renewal and drug‐resistant phenotypes. Specifically, when a Wnt ligand, such as Wnt3a, binds to the Frizzled receptor on the cell membrane, it initiates a cascade of reactions that inhibit the activity of the β‐catenin phosphorylation‐degradation complex. This intricate system includes glycogen synthase kinase‐3β (GSK‐3β), adenomatous polyposis coli protein (APC) and axin. The process of inhibition results in the buildup of β‐catenin within the cytoplasm, subsequently leading to its movement into the nucleus.^129^ Once in the nucleus, β‐catenin interacts with T‐cell factor/lymphoid enhancer‐binding factor (TCF/LEF) transcription factors,^130^ and this interaction activates a range of downstream target genes, including c‐Myc, CCND1 and ATP‐binding cassette (ABC) transporters.^131^, ^132^, ^133^ Activation of these target genes significantly enhances the proliferative capacity and drug‐efflux ability of CSCs. For example, in colorectal cancer, inactivating mutations in the *APC* gene result in the continuous activation of β‐catenin, which is closely associated with tumour recurrence. Preclinical studies have shown that the small‐molecule inhibitor ICG‐001 can significantly inhibit the tumour‐forming ability of colorectal CSCs and effectively reverse chemotherapy resistance. This is realised by blocking the interaction between β‐catenin and the transcriptional co‐activator CREB‐binding protein (CBP).^134^ Additionally, the Wnt pathway has complex interactions with immunosuppressive cells in the TME, such as regulatory T cells (Tregs) and M2‐type macrophages. These interactions further facilitate the immune escape of CSCs.^135^

### 4.2 The Notch pathway: A bridge for differentiation regulation and microenvironment adaptation

The Notch receptor plays a pivotal role in a highly conserved signalling pathway essential for development and is also associated with malignant transformation.^136^ During cancer progression, the Notch pathway primarily regulates the differentiation fate and microenvironment adaptation ability of CSCs through cell‐to‐cell contact‐dependent signal transduction. When Notch receptors (Notch1–4) bind to ligands expressed by neighbouring cells, such as Jagged1 and Delta‐like ligands, a protein‐cleavage process mediated by γ‐secretase occurs, releasing the Notch intracellular domain (NICD).^137^ The activation of the Notch pathway within CSCs has been linked to metastatic behaviour across various tumours, including those of the breast, glioma, renal and ovarian cancers. In breast cancer, bone morphogenetic protein 4 (BMP‐4) activates the Notch pathway in a Smad4‐dependent manner, promoting stemness and EMT programs.^138^ Activation of the Notch signalling pathway has also been associated with tumour resistance. In TNBC, high Notch1 expression is significantly correlated with chemoresistance and poor prognosis. γ‐Secretase inhibitors targeting Notch, such as DAPT, have effectively inhibited CSC pluripotency in preclinical models.^139^ However, these inhibitors have specific toxicities to normal stem cells, such as intestinal crypt stem cells, which greatly limits their clinical application. Currently, a new generation of selective Notch inhibitors, such as monoclonal antibodies targeting the Delta‐like ligand 4 (DLL4), is under active research and development, aiming to reduce off‐target effects and improve the safety and efficacy of treatment.^140^

### 4.3 The Hedgehog pathway: A driver of metabolic reprogramming and drug efflux

The Hedgehog signalling pathway plays a vital role in embryonic tissue pattern formation, postembryonic tissue regeneration and cancer.^141^ This pathway is abnormally activated in the CSCs of tumours such as basal cell carcinoma, pancreatic cancer and medulloblastoma.^142^, ^143^, ^144^ It enhances the survival advantage of CSCs in the TME by modulating metabolic reprogramming and the expression of drug‐transporter proteins. The specific mechanism is as follows. When the Hh (Hedgehog) ligands, such as Sonic Hedgehog, bind to the Patched receptor, it relieves the Smoothened (SMO) protein's inhibition, activating the downstream Gli transcription factors (Gli1/2/3). These activated Gli transcription factors further upregulate the expression of genes such as ABCG2 and OCT4.^145^ In pancreatic cancer, Gli1 improves the drug‐efflux ability mediated by ABCG2, leading to gemcitabine resistance in tumour cells.^107^ The SMO inhibitor Vismodegib can reverse this drug‐resistant phenotype and effectively inhibit the enrichment of CSCs.^146^ Notably, there is a positive feedback loop between the Hedgehog pathway and CAFs. Specifically, CAFs secrete Hh ligands, which activate the Hedgehog pathway in CSCs. In return, CSCs secrete cytokines such as interleukin‐6 (IL‐6) to induce the activation of CAFs. Together, they maintain a tumour‐promoting microenvironment.^147^

### 4.4 The core transcription factor network and epigenetic regulation

The core transcription network comprising OCT4, SOX2 and NANOG is an essential molecular basis for maintaining CSC pluripotency. For example, in glioblastoma, under hypoxic conditions, hypoxia‐inducible factor‐1α (HIF‐1α) can stabilise the protein level of SOX2 by inhibiting its ubiquitination and degradation process, thereby maintaining the self‐renewal ability of CSCs.^89^ In addition, epigenetic modifications, such as DNA methylation and histone acetylation, are also deeply involved in regulating CSC pluripotency. For instance, DNA methyltransferase DNMT1 can maintain the undifferentiated state of CSCs by silencing differentiation‐related genes, such as *p21*.^148^ Conversely, histone deacetylase inhibitors, such as suberoylanilide hydroxamic acid (SAHA), can effectively inhibit CSC pluripotency by restoring the expression of tumour‐suppressor genes.^149^, ^150^

### 4.5 The STAT3 and NF‐κB pathways: Hubs for immune evasion and inflammatory microenvironment

The persistent activation of signal transducer and activator of transcription 3 (STAT3) enhances the immune evasion of CSCs by increasing the levels of various factors, including PD‐L1 and IL‐6.^109^ In liver cancer, STAT3 and the Wnt pathway act synergistically to promote the chemoresistance of CSCs by activating c‐Myc.^151^ The NF‐κB signalling pathway primarily regulates inflammatory processes, evident in pathways such as tumour necrosis factor‐α (TNF‐α) and interleukin‐1β (IL‐1β), thereby establishing a favourable microenvironment for the persistence of CSCs.^152^ In breast cancer CSCs, NF‐κB facilitates the metastatic potential of CSCs by inducing the EMT phenotype.^153^ IκB kinase inhibitors can notably inhibit the activity of CSCs.^154^

### 4.6 Therapeutic strategies and challenges of targeting signalling pathways

Targeted drugs developed against the above‐mentioned signalling pathways, such as the Wnt inhibitor LGK974 and the Hedgehog inhibitor Glasdegib, have gradually entered the clinical trial stage.^155^, ^156^ However, in practical applications, these drugs face numerous challenges. First, a compensatory activation phenomenon occurs among different signalling pathways, often leading to drug resistance in tumour cells. Second, these drugs have specific toxicities to normal stem cells, restricting their clinical dosage and application range. Third, the existence of tumour heterogeneity extremely limits the therapeutic effect of single‐target drugs. To overcome these bottlenecks, combination therapy strategies, such as combining Wnt inhibitors and immune checkpoint blockers.^157^ and precision typing therapy based on single‐cell sequencing technology, are expected to become future development directions. In addition, interventions targeting epigenetic regulation (e.g., the EZH2 inhibitor Tazemetostat^158^) and targeting metabolic reprogramming (e.g., inhibition of glycolysis) have provided new ideas and strategies to eradicate CSCs.

The maintenance of CSC pluripotency depends on the coordinated regulation of multiple signalling pathways. Targeting these key signalling nodes can effectively inhibit the malignant biological behaviours of CSCs. To achieve long‐term effective control of cancer and overcome challenges such as tumour heterogeneity and treatment resistance, it is necessary to comprehensively consider multiple aspects, including microenvironment remodelling, immune regulation and epigenetic intervention, and develop multi‐level combination treatment regimens.

## 5 INTERACTION BETWEEN CSCS AND THE TUMOUR MICROENVIRONMENT

**FIGURE 3:**
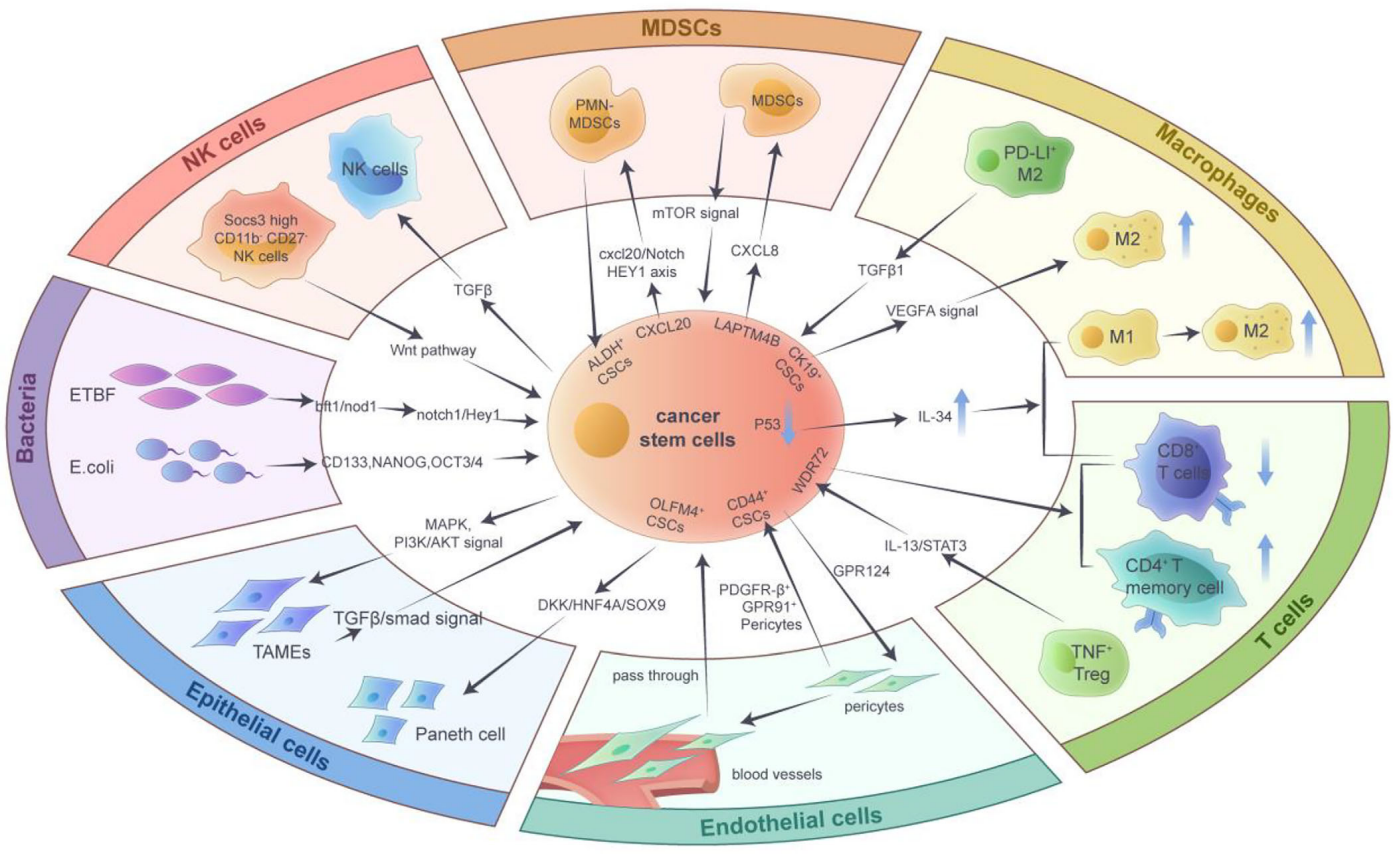
Interaction of cancer stem cells (CSCs) with the tumour microenvironment. Complex interactions exist between CSCs and other components (including myeloid‐derived suppression of cell [MDSCs], macrophages, T cells, endothelial cells, epithelial cells, natural killer [NK] cells and microbiota) in the tumour microenvironment. MDSCs can lead to the emergence of CSCs and play a key role in regulating programmed death‐ligand 1 (PD‐L1) expression. And tumour‐associated macrophages (TAMs) polarised to the M2 phenotype secrete transforming growth factor‐beta [TGF‐β]) and interleukin‐10 (IL‐10), which promote CSCs survival. T cells are suppressed by CSC‐derived PD‐L1 and IL‐34, which drive Treg expansion and CD8^+^ T cell depletion. Moreover, pericytes support the self‐renewal of CSCs through methionine secretion and GPR91 signalling. Also, Paneth cells in colorectal cancer create an ecological niche for OLFM4^+^ CSCs through DKK2/HNF4A‐SOX9 axis activation. In addition, microbiota induces genomic instability and marker expression of CSCs.

The TME is composed of multiple cell types and factors produced and secreted by these cells. These various components work together to drive biological processes such as initiation, expansion, invasion and drug resistance of tumour cells and CSCs.^159^ The heterogeneity of cells within the TME is a key factor driving cancer progression. Among these interactions, the complex interactions between CSCs and immune and non‐immune cells in the TME are gaining increasing attention from researchers.^160^, ^161^ Recent studies have indicated that the interactions of multiple cell types, such as tumour‐associated myoepithelial cells (TAMEs), Paneth cells, pericytes, T cells, macrophages, MDSCs, NK cells and microbiota, can determine the differentiation of CSCs^162^ (Figure 3).

### 5.1 CSCs and tumour‐associated myoepithelial cells

Under normal conditions, myoepithelium can inhibit cancer growth and slow invasive ductal carcinoma invasion. However, a growing number of studies have found that TAMEs can activate the TGFβ/Smads signalling axis in invasive ductal carcinoma cells through the production of TGFβ1. This activation subsequently promotes EMT, increases the stemness phenotype and exacerbates cancer invasion and migration.^163^ TAMEs are a group of cells that play a crucial role in the TME. They originate from normal epithelial cells adjacent to the tumour or from CSCs that differentiate into epithelial cells, and are often considered part of the tumour‐associated epithelium.^164^ In breast cancer, CSCs can initiate TAMEs, promote the differentiation of breast epithelial cells, and accelerate the transformation to more aggressive cancer types. This process is accompanied by the activation of MAPK and PI3K/AKT signalling pathways.^165^

### 5.2 CSCs and Paneth cells

Paneth cells are usually found in the intestinal epithelium and are detected in other organs within the digestive tract. These cells regulate the formation of CSC ecological niches and influence the malignant progression of cancer.^166^, ^167^ Sakahara et al. isolated tissues from advanced colorectal cancer patients for organoid culture and found that OLFM4^+^ CSCs first produce secretory cells, followed by the expansion of organoids with Paneth‐like cell characteristics. OLFM4^+^ stem cells can directly generate Paneth‐like cells and promote cancer progression by regulating the tumour niche.^168^ In mouse models, adenoma tissues with increased Paneth cells showed higher angiogenesis and higher expression of EphB2, a stem cell marker gene. Kaplan–Meier analysis showed that colorectal adenocarcinoma patients with positive Paneth cells had significantly reduced survival times compared to those with negative Paneth cells. This indicates that Paneth cells can create a CSC microenvironment and play an important role in the initiation and early progression of cancer.^169^ Furthermore, Paneth cells generate a stem cell niche for cancer and are regulated by the *DKK2* gene during cancer metastasis. Tumour‐derived organoids with *DKK2* knockout were injected into the spleen to construct a liver metastasis mouse model. The results showed that the expression of Paneth cell marker genes was significantly reduced, and the degree of liver metastasis of colorectal cancer was alleviated. Single‐cell sequencing data further revealed that the downstream target protein HNF4A of DKK2 can combine with the promoter fragment of SOX9, thus affecting the formation of Paneth cell characteristics.^170^

### 5.3 CSCs and pericytes

Pericytes, which interact closely with other cells such as vascular endothelial cells, are the mural cells of blood vessels and have been implicated in the progression of lesions such as cancer.^171^ Pericytes can be regarded as an integral component of the TME, regulating the movement of microvessels, creating a pre‐metastatic niche and influencing tumour cell growth and drug resistance through paracrine‐dependent effects.^172^, ^173^, ^174^ Recent studies have identified a subpopulation of pericytes that highly express PDGFR‐β and GPR91. Succinate derived from tumour cells can interact with GPR91 on pericytes, inducing autophagy and promoting methionine production. The use of GRP91‐targeted inhibitors can specifically target PDGFR‐β^+^ GPR91^+^ pericytes, thereby reducing methionine production, inhibiting self‐renewal of CSCs and enhancing chemotherapy sensitivity in clear cell renal cell carcinoma (ccRCC).^13^ Moreover, in lung adenocarcinoma, CD44^+^ lung CSCs produce a large number of vascular pericytes and promote the migration of endothelial cells, inducing brain metastasis by regulating the expression of GPR124. CSC‐derived pericytes (cd‐pericytes) can pass through and enter the blood vessels, colonise the brain through blood circulation and promote the formation of CSCs.^74^ Additionally, recent studies have found a significant negative correlation between the recruitment of tumour perivascular cells and the prognosis of glioma patients. Eliminating pericytes derived from glioma stem cells can disrupt the blood‐tumour barrier, promote vascular permeability and enhance the chemotherapy efficacy of drugs, thus making it feasible to target pericytes to alleviate chemotherapy resistance in brain tumours.^175^

### 5.4 CSCs and T cells

In multiple cancer types, CSCs modulate the effects of cytotoxic T lymphocytes by producing cytokines and chemokines.^176^ Researchers have found that nearly 50% of ovarian cancer tissues exhibit upregulation of PD‐L1 expression, while over 80% show high CD8^+^ and CD4^+^ T cells. PD‐L1 is closely related to the expression of stem cell marker genes *CD44* and *LGR5* in ovarian cancer and is involved in tumour recurrence.^110^ Also, the inactivation of transcription suppressor gene *p53* can promote the secretion of IL‐34 by CSCs, exacerbating the increase in CD36‐induced fatty acid oxidation metabolism. This process promotes the transformation of TAMs from the M1 to M2 type, thereby hindering the anti‐tumour effect of CD8^+^ T cells.^177^ Using mRNA sequencing and single‐cell sequencing data from the TCGA database, WDR72 was identified as a gene associated with CSCs. Patients with high expression of WDR72 have a poor prognosis, accompanied by a significant decrease in PD‐L1 expression in tumour tissue. Meanwhile, the increased expression of the *WDR72* gene is negatively correlated with the production of CD8^+^ T cells and the activation of CD4^+^ T memory cells.^111^ Furthermore, regulatory T (Treg) subpopulations in tumour tissues also play an important role in regulating CSC survival and expansion. Researchers collected gastric cancer and adjacent tissues and conducted single‐cell sequencing. Through bioinformatics analysis of the sequencing results, they found that TNF‐α^+^ Tregs can be recruited into tumour tissues and affect patient survival. Further mechanistic exploration revealed that TNF‐α^+^ Tregs regulate the IL‐13/STAT3 signalling axis, inhibiting tumour stemness and progression.^178^

### 5.5 CSCs and macrophages

Tumour‐associated M2‐type macrophages play a key role in regulating CSC biological processes such as tumour growth, invasion, metastasis and drug resistance.^179^ IL‐33 in large oncosomes produced by SCC stem cells can create a TME rich in TGF‐β, maintain stem cell self‐renewal and evade apoptosis by inducing the antioxidant process of NRF2. Mechanistically, NRF2 promotes the expression of ATG9B and the loading of ANXA1, thereby inducing AXNA1^+^ precursor myeloid cells to become immunosuppressive macrophages. The inhibition of ATG9B activity or the promotion of ANXA1 depletion can reduce macrophage production and slow down tumour progression.^14^ In addition, researchers conducted single‐cell sequencing analysis on tissues from 11 HBV‐related liver cancer patients, including 64 581 human liver cancer cells and adjacent cells. The results showed that CK19^+^ CSCs expressed liver CSC markers and were negatively correlated with patient prognosis. In CK19^+^ HCC, TAM subpopulations with M2‐like features (SPP1^+^ TAMs) are enriched and promote tumour migration by activating VEGFA signalling.^180^ Through spatial transcriptome analysis of hepatocellular carcinoma patients and mouse model tissues, it was found that PD‐L1^+^ M2 macrophages can highly express TGF‐β1 and maintain the survival of CSCs, leading to the recurrence of minimal residual disease‐related liver cancer. In two mouse models, using PD‐L1 and TGF‐β inhibitors to clear M2 macrophages can activate effector T cells, inhibit the expansion of CSCs and reduce cancer recurrence.^181^

Microglia, also known as TAMs, have the highest proportion of components in the brain TME through single‐cell sequencing technology. CSCs reshape the phenotype of macrophages through direct contact and secretion of cytokines, promoting their transformation into immunosuppressive type (M2 type).^98^, ^182^ In lung cancer brain metastases, TAMs are highly enriched and regulate complement‐induced synaptic pruning, activate microglia and clear neutrophils.^183^

### 5.6 CSCs and MDSCs

MDSCs can lead to the emergence of CSCs and play a key role in regulating PD‐L1 expression. In ovarian cancer, MDSCs can increase the number of CSCs with high ALDH expression by promoting the production of PGE2 while regulating the mTOR signalling pathway to increase the proportion of cells expressing PD‐L1. Targeted inhibition of CSC expansion and tumour PD‐L1 expression can deplete MDSCs and hinder the progression of ovarian cancer.^184^ The tumour stemness marker ALDH1A1 plays a vital role in tumour initiation, progression and recurrence. ALDH1A1 can regulate the pH in tumour cells, activate the production of phosphorylated TAK1, upregulate the NF‐κB signalling pathway and cause the expansion of MDSCs, thus forming an immunosuppressive microenvironment and inducing the malignant progression of breast cancer.^185^ Moreover, MDSCs and CSCs jointly drive immune suppression, drug resistance and tumour recurrence in the glioma microenvironment. In an in vitro co‐culture model, it was found that CSCs derived from patients can induce the formation of MDCS‐mediated immunosuppressive microenvironment in gliomas by secreting macrophage migration inhibitory factor (MIF).^186^ Observations in glioma animal models indicated that the expression of G9a can enhance the influx of IFN‐γ^+^CD4^+^ and CD8^+^ T cells, while simultaneously diminishing the entrance of PD‐1^+^CD4^+^ T cells, MDSCs and M2 macrophages within the TME. This process affects the stemness of glioma stem cells and delays tumour progression.^187^ *LAPTM4B*, an important gene regulated by transcription factor ETV1, can affect the self‐renewal of liver CSCs by modulating the Wnt1/c‐Myc/β‐catenin signalling axis. Meanwhile, it can activate the migration of MDSCs by increasing the production of CXCL8, thereby exacerbating tumour deterioration.^188^ Additionally, CCL20 can affect the self‐renewal of CSCs in breast cancer by regulating TME components. Polymorphonuclear (PMN)‐MDSCs can be recruited in the immune microenvironment of in situ tumours. Further research has found that PMN‐MDSCs recruited by cancer cells overexpressing CCL20 can synthesise and secrete CXCL2, subsequently upregulating the CXCR2/NOTCH1/HEY1 signalling axis and increasing the number of ALDH^+^ breast CSCs.^189^

### 5.7 CSCs and NK cells

NK cells are a class of innate immune cells with specialised functions to recognise and kill abnormal, infected cells and tumour cells.^190^, ^191^ A few types of CSCs can influence tumour malignant progression by modulating the killing activity of NK cells. Through mass spectrometry detection and single‐cell sequencing analysis of primary glioma tissues, it was found that glioma stem cells can evade immune surveillance by NK cells. Glioma stem cells can use αv integrin to induce high expression of the TGF‐β pathway, thereby achieving direct contact with NK cells and exacerbating tumour growth and migration.^192^ In TNBC, Socs3^high^CD11b^−^CD27^−^ NK cells represent an immature subtype. These cells can reduce the toxicity of granzyme, activate the expression of the Wnt signalling pathway and enhance the expansion ability of CSCs, thereby aggravating cancer progression in mice.^193^ To explore the relationship between NK cells and the proliferation of liver CSCs, researchers used flow cytometry to select CSCs with EpCAM^low^ and EpCAM^high^ characteristics and then co‐cultured the sorted cells with NK cells in vitro. The results showed that the cancer recurrence rate of patients with high EpCAM expression was significantly upregulated and mediated their tolerance to NK cell killing. Blocking the expression of CEACAM1 on the surface of EpCAM^high^ cells using CEACAM1 antibodies can increase the toxicity of NK cells and promote tumour regression.^86^

### 5.8 CSCs and microbiota

In recent years, extensive research has shown that gut microbiota and tumour‐resident microorganisms also play important roles in regulating the functions of various CSCs.^194^, ^195^ A portion of gut microbiota can affect CSCs in various tumours, including colorectal cancer and melanoma. Using RNA sequencing to analyse circRNA and microRNA (miRNA) in antibiotic‐treated and untreated B16F10 mouse metastatic tumour models, researchers found that gut microbiota can inhibit the level of mmu_circ_0000730 by regulating IL‐11 expression. And subsequent molecular experiments demonstrated that mmu_circ_0000730 inhibits the EMT process and stemness of CSCs by competitively binding to mmu‐miR‐466i‐3p.^196^ In addition, colorectal cancer patients often harbour a large number of *E. coli* bacteria that secrete colistin in their intestines. These *E. coli* strains can induce genomic instability and gene mutations, and promote the expression of CSC markers such as CD133, NANOG and OCT4. The subsequent increase in CSCs leads to chemotherapy tolerance by increasing the EMT in colorectal cancer patients, thus intensifying the malignancy of the disease.^197^ There is also a small portion of tumour‐resident microorganisms that regulate CSCs and tumour progression. By analysing and evaluating the microbial composition of breast cancer tissues, researchers found that *enterotoxigenic Bacteroides fragilis* (*ETBF*) is significantly enriched in chemotherapy‐insensitive tissues. Mechanistically, *ETBF* produces a toxic protein, bft‐1, that directly binds to Nod1. In ALDH^+^ breast CSCs, the expression of Nod1 is upregulated, and the Notch1/Hey1 signalling axis is stimulated to promote the survival and expansion of breast CSCs.^198^ In addition, *Helicobacter pylori* in the gastric mucosal microenvironment can activate epithelial–mesenchymal transformation while maintaining gastric stem cell‐like characteristics and increasing the number of CSCs.^199^

### 5.9 CSCs and other components

The brain TME serves as the core ecological niche for the survival, self‐renewal and drug resistance of CSCs, and its cellular composition and signalling interactions jointly shape the malignant biological processes of CSCs. Brain endothelial cells can enhance the adhesion between CSCs and blood vessels, thereby strengthening the ability of tumour invasion and metastasis. CEMIP is a tumour stemness‐related protein that is upregulated in tissues and exosomes of patients with brain metastases. It can transport brain endothelial cells through extracellular vesicles and induce endothelial cell branching, reshape cerebral blood vessels and promote brain metastasis by activating pro‐inflammatory cytokines (including TNF, PTGS2, CXCL, etc.).^200^ Moreover, astrocytes represent an essential cell type within the glioblastoma microenvironment, significantly influencing the malignant progression of tumours driven by CSCs.^201^ Researchers extracted extracellular vesicles released by glioma stem cells and added them to the culture medium of normal human astrocytes. The results showed that the extracellular vesicles secreted by stem cells could induce the proliferation, elongation and drug resistance of human astrocytes by targeting the miR‐3065‐5p/DLG2 signalling pathway.^202^

Furthermore, besides the regulatory roles played by different cells within the TME on CSCs, soluble elements such as growth factors, cytokines and chemokines also influence the self‐renewal capabilities of CSCs. The release of the growth factor TGF‐β has a strong correlation with the self‐renewal process of CSCs, as increased levels can markedly boost the phosphorylation of Smad2/3, simultaneously stimulating the expression of stem‐related genes such as SOX2 and OCT4.^203^ Additionally, the growth factor HGF secreted by pancreatic stellate cells activates the expression of c‐MET, leading to the nuclear translocation of YAP and stabilising the structure of HIF‐1α, which promotes the tumour cell spheroidisation ability and upregulates the expression of stemness marker genes NANOG and OCT4.^204^, ^205^ Moreover, the expression of IL‐6 is significantly elevated in CSCs of prostate cancer patients. The use of IL‐6 antibody (cetuximab) can effectively inhibit the appearance of stem cell‐like features in patients with malignant tumours and better resist tumour proliferation.^206^, ^207^ Furthermore, the chemokine CCL2 in the TME can promote the nuclear translocation of β‐catenin and exhibit characteristics of CSCs by activating the PI3K/AKT signalling pathway.^208^ Also, the absence of chemokine CXCL12 can inhibit p38 signalling transduction in AML cells and promote the sensitivity of leukaemia stem cells to chemotherapy drugs, maintaining a stable microenvironment.^209^

Extracellular vesicles (including exosomes and microvesicles) are also important carriers connecting CSCs and their surrounding TME. It can load molecules such as nucleic acids and proteins, thereby mediating cell–cell interactions and reshaping the TME. Bladder CSCs‐derived exosomes can deliver nucleic acid LUCAT1, which increases the expression of stemness‐related marker genes by stabilising the expression of HMGA1 mRNA and promoting the development of chemotherapy resistance in bladder cancer.^210^ In addition, researchers found that the exosomes secreted by pancreatic CSCs were rich in miR‐210, which was then taken up by macrophages and promoted the polarisation of M2 macrophages by inhibiting the expression of FGFRL1.^211^

## 6 FUNCTIONS OF CSCS IN TUMOUR DEVELOPMENT

In recent years, it has been found that a major factor contributing to the recurrence of cancer in patients after initial radiation and chemotherapy is the emergence of tumour dormant cells, which are often associated with the presence of CSCs.^212^ CSCs in the TME are considered key factors in the initiation, progression, and metastasis of tumours and are closely related to cancer‐related mortality.^213^ The complex interactions of CSCs with a variety of biological molecules, including cells, chemokines and exosomes, in the TME influence tumour cell proliferation, metastasis, drug resistance, autophagy and immune escape^214^, ^215^ (Table 2).

**TABLE 2:** Functions of cancer stem cells (CSCs) in tumour development.

Function	Cancer type	Marker genes/cells	Downstream target	References
Tumour growth and metastasis	Wilms tumour	SIX2^+^CITED1^+^ cells	Integrins ITGβ1 and ITGβ4	^216^
	Colorectal cancer	FAK	CD133, CD44, Nanog, OCT4, c‐Myc	^217^
	Prostate cancer	NUMB	miR‐9‐5p	^75^
	Nasopharyngeal carcinoma	stem‐cell‐like tumour cells	PCK2, ACSL4	^218^
	Osteosarcoma	WNT5B	HYAL1 and SOX2	^167^
Angiogenesis	Acute myeloid leukaemia	IL‐5	VEGF signal	^219^
	Meningiomas	Notch3^+^ stem cells	Notch3	^220^
	Glioma	OLFML3	POSTN, TBK1 signal	^221^
Autophagy	Liver cancer	CircHULC	TP53INP2/DOR and LC3	^222^
	Glioma	LAMP2A	CMA	^223^
	Ovarian cancer	TFEB, trehalose	OCT4 and LAMP2A	^224^
	Head and neck cancer	FOXO3	SOX2	^133^
Metabolism	Breast cancer	MIF	WNT/β‐catenin	^225^
	Glioma	CD47	lactate and induce histone lactylation	^226^
	Glioma	PDGF	N6‐methyladenosine	^227^
	Pancreatic cancer	ISG15	ISGylation	^228^
Immune escape	Breast cancer and colorectal cancer	PD‐L1	Frizzled 6, β‐catenin	^229^
	Oesophageal cancer	QSOX1	CD8^+^ T cells	^230^
Chemoresistance	Oral cancer	CD44^+^ cells	NRF2 signal	^76^
	Breast cancer	CD96	Src‐Stat3‐Opa1 signal	^231^

### 6.1 CSCs in tumour growth and metastasis

CSCs exploit alterations in multiple molecular pathways to promote tumour growth, invasion, migration and metastasis. Through analysis of single‐cell RNA sequencing and spatial transcriptome data, researchers have confirmed that SIX2^+^CITED1^+^ cells, known as renal CSCs, are capable of self‐renewal and are regulated by integrins ITGβ1 and ITGβ4. They have also proposed that changes in renal spatial transcriptome expression profiles are important factors driving the progression of Wilms tumour.^216^ Moreover, FAK is upregulated in colorectal cancer and can be activated through phosphorylation to participate in the proliferation of CSCs. The use of FAK inhibitors or AKT inhibitors can reduce the expression of CSC biomarkers, including CD133, CD44, NANOG, OCT4, c‐Myc and so forth, and limit CSC‐like features and colorectal cancer metastasis.^217^

Additionally, previous studies have shown that NUMB is an inhibitor of prostate CSCs. It can affect the number of CD44^+^ prostate CSCs by regulating the expression of miR‐9‐5p, thereby inhibiting cancer migration and invasion.^75^ CSC‐like tumour cells can inhibit ferroptosis sensitivity and induce resistance to chemotherapy and radiotherapy. In‐depth mass spectrometry studies have found that CSC‐like tumour cells regulate the phosphorylation modification of the mitochondrial metabolism‐related kinase PCK2 and the expression of the *ACSL4* gene, thereby promoting phospholipid remodelling associated with ferroptosis and facilitating distant metastasis of cancer.^218^ Through analysis of osteosarcoma cell lines and stem cell spheres isolated from patients, it was found that WNT5B is highly expressed in osteosarcoma stem cells. Meanwhile, the significant upregulation of WNT5B in stem cells can promote the possibility of osteosarcoma metastasis to the lungs and liver by regulating the levels of HYAL1 and SOX2.^232^

### 6.2 CSCs in tumour angiogenesis

CSCs are able to exacerbate cancer progression by altering the degree of angiogenesis in tumour cells. In AML patients, leukaemia stem cells exist in a quiescent state and play a crucial role in cancer recurrence. Through analysis of patient xenograft model samples, it was found that leukaemia stem cells upregulate the IL‐5 and VEGF signalling pathways, maintaining the expansion of CSCs.^219^ Reports have identified a group of Notch3^+^ stem cells around blood vessels that can promote tumour initiation, enhance cell expansion ability and intensify tumour‐related angiogenesis. This process promotes the malignant progression of meningiomas and exacerbates resistance to radiotherapy.^220^ In addition, glioma stem cells can regulate the progression of gliomas and maintain key markers of stem cells. In glioma stem cells, the circadian rhythm‐related OLFML3 plays a crucial role in the upregulation of HIF‐1α, activated POSTN. Subsequently, the secretion of POSTN outside the cell promotes the activation of the TBK1 signalling axis in endothelial cells. It accelerates the process of tumour angiogenesis, which elucidates the molecular mechanism of the interaction between tumours and endothelial cells to exacerbate cancer.^221^

### 6.3 CSCs in tumour autophagy

Several genes and drugs can influence the survival and expansion of CSCs by modulating the activity of autophagy‐related genes and signalling pathways. For instance, CircHULC promotes the binding of TP53INP2/DOR to LC3 and enhances the interaction between LC3 and ATG proteins (including ATG3 and ATG12, etc.). As a result, CircHULC increases the production of autophagosomes, enhances the self‐renewal ability of liver CSCs and exacerbates the malignant progression of cancer cells.^222^ Chaperone‐mediated autophagy (CMA) is more prevalent in glioma stem cells and can induce their expansion. Downregulating the autophagy marker LAMP2A promotes apoptosis of CSCs and inhibits their self‐renewal, thereby alleviating tumour malignancy in in vivo experiment.^223^ In ovarian CSCs, fructose metabolism mediates the CMA pathway and regulates cancer progression. The transcription factor TFEB and trehalose can upregulate CMA molecular markers OCT4 and LAMP2A levels. Knocking down the LAMP2A gene inhibits the spheroidisation of ovarian CSCs and activates the expression of GLUT5.^224^ Moreover, researchers have explored the association between autophagy and head and neck CSCs in adverse environments. Their findings indicate that autophagy inhibitors, such as chloroquine and 3‐MA, can reduce the stem cell characteristics induced by starvation or hypoxia. Mechanistically, the autophagic substrate FOXO3, which is enriched in cells with low autophagy levels, can directly bind to the promoter sequence of SOX2. This interaction reduces SOX2 levels and restrains the self‐renewal of CSCs in both in vivo and in vitro experiments.^133^

### 6.4 CSCs in tumour metabolism

Cancer development and the monitoring function of the immune system are deeply influenced by metabolic reconfiguration. Exosomes derived from hypoxic breast cancer cells and breast CSCs induce the readjustment of the glycolysis pathway in breast cancer cells. MIF, a key factor in breast CSC exosomes, promotes the increase of glycolytic enzyme aldolase C by activating the WNT/β‐catenin signalling pathway, thereby enhancing glycolysis.^225^ Additionally, glioma stem cells and microglia derived from patients can produce lactate and induce histone lactylation, enabling tumour cells to acquire metabolic‐mediated epigenetic activation modifications. This immunosuppressive program increases immune escape and promotes tumour progression by activating CD47 expression in glioma cells.^226^ Moreover, the PDGF signalling axis can induce the upregulation of oncogenic N6‐methyladenosine in glioma cells, thereby maintaining the growth and self‐renewal of CSCs by regulating cancer metabolism.^227^ Various stem cells are involved in processes such as cancer initiation, migration, metabolism and drug resistance. Theoretically, clearing the stem cell population can inhibit tumour metastasis and recurrence, but the strong plasticity of stem cells limits this approach's effectiveness. Pancreatic CSCs can promote the expression level of ISG15 and induce increased protein ISGylation in mitochondria to maintain mitochondrial function, regulate metabolic plasticity and further drive CSC proliferation.^228^

### 6.5 CSCs in immune escape and chemoresistance

CSCs significantly contribute to driving biological processes, including tumour cell immune escape and drug resistance. PD‐L1 can promote the survival and expansion of CSCs through novel molecular mechanisms, resulting in immune escape. PD‐L1 directly binds to Frizzled 6, activating β‐catenin signalling, increasing targeted gene expression and blocking tumour progression. Moreover, β‐catenin protein can reciprocally regulate PD‐L1, forming a positive feedback loop between PD‐L1 and β‐catenin, thereby promoting CSC survival and exacerbating cancer progression.^229^ Furthermore, dormant CSCs are instrumental in immune evasion and chemotherapy resistance, which are important factors leading to cancer recurrence and deterioration. In the TME of oesophageal SCC, QSOX1 is highly expressed and facilitates the clearance of CD8^+^ T cells. Researchers have used ebselen in combination with anti‐PD‐1 therapy and chemotherapy to suppress QSOX1 expression. This approach downregulates PD‐L1, enhances CD8^+^ T cell infiltration and clears CSCs.^230^

CSCs influence the generation of resistance to chemotherapeutic agents by regulating multiple biological processes. Researchers have found that autophagy, which relies on the action of ATG5 and BECN1, plays a vital role in preserving cancer stemness and mitigating the cytotoxic effects induced by cisplatin. CD44^+^ cells that are deficient in autophagy exhibit decreased levels of reactive oxygen species and boost cancer stemness through the activation of NRF2 signalling. Inhibiting both autophagy and NRF2 signalling simultaneously can increase the cytotoxic effects of cisplatin, diminish the proliferation of oral CD44^+^ cells and provide new possibilities for addressing chemoresistance and tumour recurrence related to oral cancer CSCs in clinical practice.^76^ In addition, the expression of CD96 in breast cancer tissue samples has been found to increase significantly and is closely related to patient prognosis. In xenograft tumour models, reducing CD96 levels can accelerate mitochondrial fatty acid β‐oxidation by regulating the Src‐Stat3‐Opa1 signalling axis, thereby enhancing the resistance of breast CSCs to chemotherapy.^231^

## 7 CLINICAL STRATEGIES TARGETING CSCS

### 7.1 CSCs in diagnosis

Identifying subpopulations of CSCs with strong drug resistance and tumourigenicity can help develop new diagnostic and therapeutic targets. Recent research employing single‐cell RNA sequencing techniques has shown a heightened expression of NNMT during the malignant advancement of cardia adenocarcinoma. NNMT can activate the WNT signalling pathway and promote the survival of AQP5^+^ stem cells by regulating nicotinamide metabolism. Through heterogeneity analysis of cardia adenocarcinoma tissue, researchers have found that cancer patients with NNMT^+^/AQP5^+^ phenotypes are more likely to experience poorer malignant progression. These findings hold important implications for tumour diagnosis and therapy.^233^

### 7.2 CSCs in prognostic prediction

Specific subpopulations of CSCs can serve as markers for prognostic prediction in cancer patients. A cohort study and flow cytometry analysis of 127 gastric cancer patients showed that CD24^+^CD44^+^CD54^+^ EpCAM^+^ cells were positively correlated with tumour metastasis and were present in untreated patients. Quantitative analysis of this cell population displays significant clinical value in prognosis prediction and may potentially serve as a biomarker for prognosis.^87^ Additionally, researchers have used spatial transcriptomics techniques to explore the heterogeneity of primary and metastatic colorectal tumours. The results showed a significant increase in the number of colorectal CSCs in metastatic tumours and revealed interactions between CD74‐MIF and metastatic tumour tissue. The in‐depth analysis identified FOXD1 as a marker gene for colorectal CSCs, which can predict cancer patient survival and is of significant clinical value.^234^

Several key genes that influence the expansion of CSCs play a potential role in predicting the effectiveness of immunotherapy. B3GNT5 has been elucidated to be important in increasing tumour‐associated T cell infiltration. Analysis of RNA sequencing results in the TCGA database shows that B3GNT5 is associated with poor prognosis in various cancer types and is highly correlated with T cell production and activation. Subsequent experimental results suggest that inhibiting B3GNT5 can limit the ability of stem cells to form spheres and expand, thereby reducing tumourigenicity in pancreatic cancer. These findings clarify that B3GNT5 has the potential to become an important factor in the prognosis of tumour immunotherapy.^235^

### 7.3 CSCs in tumour therapy

**FIGURE 4:**
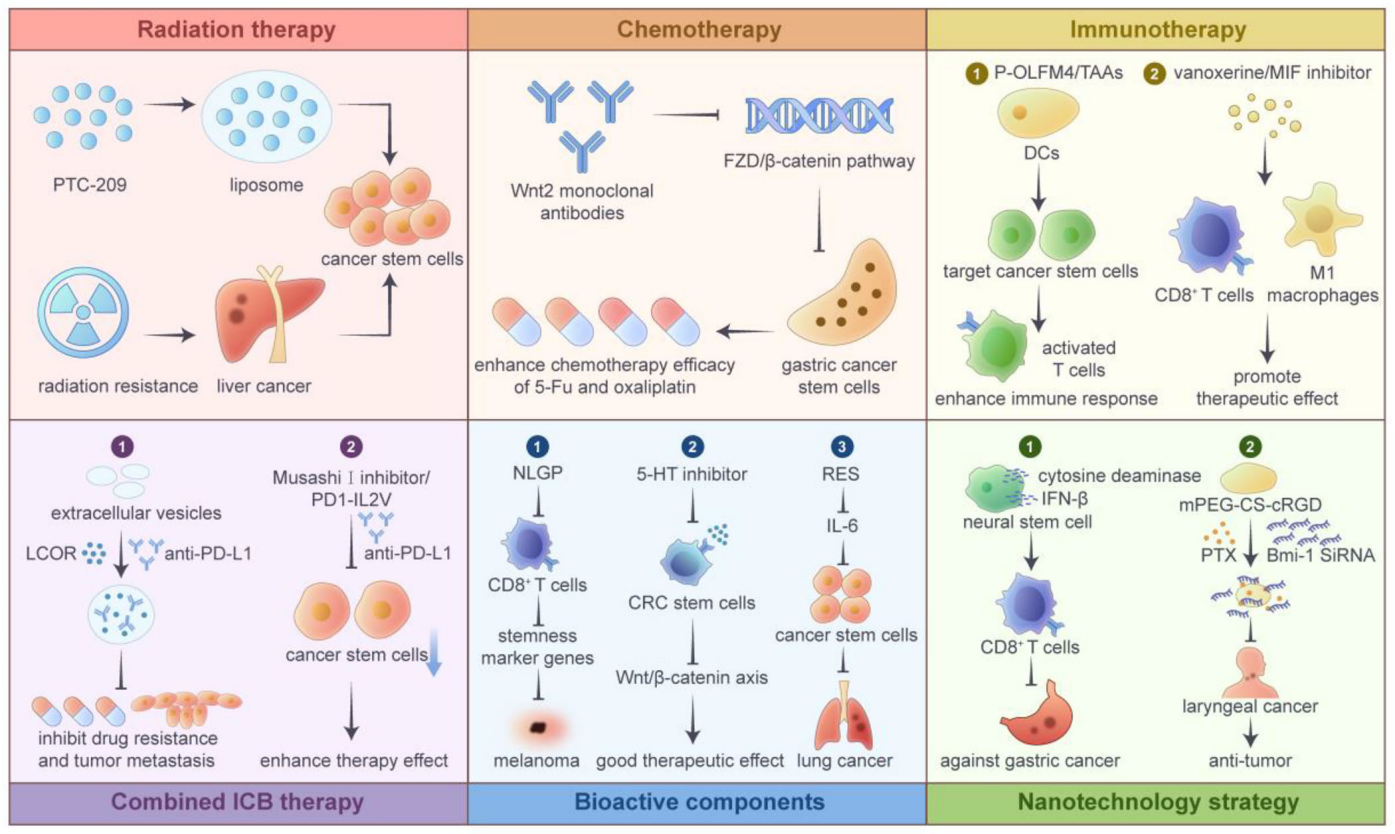
Clinical therapeutic strategies for targeting cancer stem cells (CSCs). The targeted therapy strategies for CSCs mainly include the following aspects: 1. Targeting cancer stem cells that are resistant to radiotherapy can restore the sensitivity of tumour tissue to radiotherapy; 2. The combination of CSCs expansion‐related inhibitors and chemotherapy drugs can effectively improve the efficacy of tumour therapy; 3. Due to the resistance of CSCs to immunotherapy, dendritic cell (DC) vaccines targeting CSCs markers such as Olfactomedin 4 (OLFM4) can be designed to enhance antigen delivery and immune response; 4. The combination immune checkpoint blockade (ICB) treatment strategy combines programmed cell death‐1 (PD‐1)/programmed death‐ligand 1 (PD‐L1) checkpoint inhibitors with CSCs‐specific vaccines (DC‐P‐OLFM4) or engineered cytokines (PD1‐IL2v); 5. Many bioactive ingredients are also used to target CSCs, such as neem leaf glycoprotein (NLGP), which can restore stemness enhancement induced by CD8^+^ T cell exhaustion; 6. Nanotechnology enhances drug delivery to CSCs. The challenges include overcoming compensatory pathway activation, metabolic plasticity and toxicity to normal stem cells. Future directions emphasise multi‐omics‐guided precision therapy and microenvironmental remodelling.

Currently, many therapeutic strategies have been developed to target CSCs (Figure 4 and Table 3). Radiation therapy can promote the formation of CSCs and induce tumour invasion and migration. The *BMI1* gene is an important factor in promoting radiation resistance and exacerbating poor prognosis in liver cancer patients. Encapsulating PTC‐209, a BMI1 inhibitor, in liposomes and delivering it to liver cancer tissues with radiation resistance can restore the sensitivity of liver cancer cells to radiation therapy.^236^

**TABLE 3:** Clinical strategies for targeting cancer stem cells (CSCs).

Clinical strategy	Target/drug	Cancer type	Pathway	Function	References
Diagnosis target	NNMT	Cardia adenocarcinoma	WNT	Promote AQP5^+^ stem cells	^233^
Prognostic prediction	CD24^+^CD4^+^CD54^+^EpCAM^+^ cells	Gastric cancer	–	Correlated with tumour metastasis	^87^
	FOXD1	Colorectal cancer	CD74‐MIF	Interaction between CD74‐mif and metastatic tumour tissue	^234^
	B3GNT5	Pancreatic cancer	–	Promote t cell production and activation	^235^
Therapeutic strategy	PTC‐209	Liver cancer	CSC differentiation	Restore the sensitivity to radiation therapy	^236^
	CD7‐CAR‐T cells	Leukaemia	–	Effective treatment plan for cd7‐positive tumour patients	^237^
	BC19CAR‐T	Solid tumours	–	Excellent anti‐tumour activity	^238^
	CD133	Metastatic brain tumours	–	Slow down tumour growth and prolong survival	^239^
	WNT2 monoclonal antibodies	Gastric cancer	FZD8/ β‐catenin	Enhance the chemotherapy efficacy	^240^
	ABT‐888	Colorectal cancer	MMR pathway	Enhance the chemotherapy efficacy	^241^
	OLFM4	Melanoma	–	Trigger a strong immune response	^242^
	TAAs	Glioma	–	Activated T cells had stronger targeting and killing effects	^239^
	Vanoxerine	Colorectal cancer	G9a	Improve anti‐tumour immune efficacy	^243^
	MIF inhibitor	Breast cancer	–	Promote CD8^+^ T cells and M1 macrophages	^225^
	BCAT1	Breast cancer	IFN‐γ‐induced CSCs	Improved the efficacy of cancer vaccines and increased immune checkpoint blockade (ICB) efficacy	^244^
	LCOR	Triple negative breast cancer	IFN response elements	Enhance anti‐PD‐L1 therapy efficacy	^63^
	Musashi I inhibitor	Breast cancer	–	Enhance anti‐PD‐L2 therapy efficacy	^245^
	PD1‐IL2v	Pancreatic neuroendocrine cancer	–	Enhance anti‐PD‐L3 therapy efficacy	^246^
	Neem leaf glycoprotein (NLGP)	Melanoma	stemness marker genes	Increase their sensitivity to 5‐fluorouracil (5‐FU)	^247^
	5‐HT inhibitor	Colorectal cancer	Wnt/β‐catenin	Affect the self‐renewal of CSCs	^248^
	Resveratrol	Lung cancer	IL‐6	Regulate the amount of CSCs and slow down cancer progression	^249^
	IFN‐beta and cytosine deaminase	Gastric cancer	granzyme B	Activate CD8^+^ T cells and increase anti‐tumour effect	^250^
	Rebecsinib	Leukaemia	ADAR1p150	Limit the proliferation and survival of leukaemia stem cells	^251^
	BMI‐1 siRNA	Oesophageal cancer	–	Enhance the chemotherapy efficacy	^99^

Targeting CSCs using CAR‐T cell therapy has been a promising research direction in recent years.^252^ CSCs are often considered a key factor in the occurrence, metastasis, recurrence and drug resistance of tumours. The design of CAR‐T therapy targeting CSCs has the potential to eradicate ‘seed cells’. Current CAR‐T therapies targeting CSCs have made significant breakthroughs in haematological tumours.^253^ The latest study designed a treatment strategy combining CD7‐CAR‐T cells with allogeneic HSC transplantation in 10 patients with relapsed or metastatic CD7^+^ leukaemia. The results showed that this treatment strategy can effectively alleviate the condition of 10 patients, providing a safe and effective treatment plan for CD7‐positive tumour patients.^237^ Also, researchers designed a bispecific antibody (BC19CAR‐T) targeting B‐cell maturation antigen (BCMA) and CD19, and demonstrated excellent anti‐tumour activity in a mouse xenograft model. This study elucidates the feasibility, safety and efficacy of bispecific BC19CAR‐T cells in refractory multiple myeloma.^238^ Additionally, designing CAR‐T cell therapy strategies targeting solid tumours presents broad research prospects. The tumour stemness‐related genes CD133, EpCAM, CD44, L1CAM, ROR1, CD371 and so forth are the main research targets.^67^, ^254^ At present, most studies are in the early stage of clinical trials, and some targets have shown preliminary efficacy and controllable safety. Metastatic brain tumours are also a type of solid tumour, usually with poor survival after diagnosis (no more than 12 months). CD133 is a glycoprotein highly expressed in neural stem cells and a marker gene for TICs, which can exacerbate tumour recurrence, metastasis and drug resistance. Researchers used a CD133 binder to modify CAR‐T cells that target CD133 and found that it could significantly slow down tumour cell growth and prolong mouse survival after a single dose.^239^

Inhibitors of stem cell expansion‐related pathways, when combined with chemotherapeutic agents, can effectively enhance the efficacy of tumour therapy. The transcription factor SOX4 positively regulates WNT2 expression and is also regulated by the WNT2/FZD8/β‐catenin pathway, thereby forming a positive feedback loop that maintains the self‐renewal of gastric CSCs. Treatment with WNT2 monoclonal antibodies can enhance the chemotherapy efficacy of 5‐fluorouracil and oxaliplatin by disrupting the WNT2‐SOX4 positive feedback axis.^240^ Additionally, the combination of 5‐fluorouracil and ABT‐888 (a PARP inhibitor) can accumulate DNA damage and promote the inactivation of the MMR signalling pathway, thereby increasing the death of colorectal CSCs. This combination proves to be an effective strategy for tumour therapy.^241^

CSCs have resistance to various therapies, including immunotherapy. To address this issue, researchers have designed dendritic cell (DC) vaccines targeting the CSC marker Olfactomedin 4 (OLFM4) and binding to the fusion protein P‐OLFM4 to enhance antigen delivery and immune response. The DC (P‐OLFM4) vaccine can induce T cell expansion, activate cytotoxic T cells and produce interferon‐γ, triggering a strong immune response. This approach significantly alleviates tumour progression and holds promise as an effective cancer treatment.^242^ Moreover, researchers have loaded tumour‐associated antigens (TAAs) from glioma stem cells onto DCs and co‐cultured them with T cells. The results showed that activated T cells had stronger targeting and killing effects against glioma stem cells.^255^ Due to their self‐renewal and tumour initiation characteristics, CSCs can reduce tumour immunogenicity and exacerbate tumour proliferation and metastasis. Molecules targeting G9a HMTase can effectively inhibit the growth of colorectal CSCs, but their safety profile limits their clinical application. Researchers have identified vanoxerine, a dopamine transporter (DAT) antagonist with established clinical safety, as a means to downregulate G9a expression. This means it inhibits CSC activity, enhances lymphocyte infiltration and improves anti‐tumour immune efficacy.^243^ In addition, targeted inhibition of MIF in breast CSCs can promote the production of CD8^+^ T cells and M1 macrophages while reducing the number of regulatory T cells and cancer‐related neutrophils. This indicates that MIF inhibition can improve the therapeutic effect of immune checkpoint blockers and has the potential to become a new target for tumour treatment.^225^

Immune checkpoint blockade (ICB) has made significant progress in tumour therapy, but many patients remain unresponsive to this treatment. CSCs can tolerate the cytotoxicity of T cells, and the IFN‐γ produced by effector T cells can induce tumour cells to become CSCs by regulating BCAT1. Beziaud et al. improved the efficacy of cancer vaccines and increased ICB efficacy by targeting BCAT1 to inhibit IFN‐γ‐induced CSC formation.^244^ Also, the ligand‐dependent corepressor factor (LCOR), a major transcription factor in APM, binds to IFN response elements in an IFN‐independent manner. LCOR in CSCs is closely related to the efficacy of clinical ICB therapy, as it can enhance the therapeutic effect of immune blockade therapy. In preclinical models, the combination of extracellular vesicles delivering LCOR and anti‐PD‐L1 therapy can alleviate drug resistance and tumour metastasis in TNBC.^63^ Moreover, Musashi I increases the stability of oncogenic transcripts and affects the expression of stemness‐related genes, thus maintaining the proliferation of breast cancer cells. Inhibiting the expression of Musashi I can reduce the production of CSCs, which is associated with low expression of PD‐L1. The combination of Musashi I inhibitors and immune checkpoint inhibitors can control the emergence of CSCs and achieve better cancer treatment outcomes.^245^ In a mouse model of pancreatic neuroendocrine cancer, the engineered immune cytokine PD1‐IL2v can induce the infiltration of stem cell‐like effector CD8^+^ T cells, leading to tumour regression and improved survival rates. The combination therapy of anti‐PD‐L1 and PD1‐IL2v can maintain the response duration and improve therapeutic efficacy, providing a theoretical basis for clinical trials.^246^

Some bioactive components can influence the self‐renewal of CSCs and regulate tumourigenesis and progression. Neem leaf glycoprotein (NLGP) has an immune‐dependent anti‐tumour effect. It can effectively activate dormant CSCs and increase their sensitivity to 5‐fluorouracil by downregulating the stemness marker genes. In addition, NLGP treatment can restore the stemness enhancement caused by depleted CD8^+^ T cells and alleviate the progression of melanoma.^247^ Moreover, serotonin (5‐HT) produced by enteric serotonergic neurons binds to colorectal CSCs with high expression of 5‐HT receptors and activates the Wnt/β‐catenin signalling axis. In mice, inhibiting the 5‐HT axis can significantly affect the self‐renewal of CSCs and demonstrate favourable therapeutic effects on colorectal cancer.^248^ Resveratrol (RES), a chemical compound extracted from plants, has anti‐tumour activity. It slows down lung cancer progression by regulating the amount of lung cancer stem‐like cells and IL‐6 expression in the TME.^249^

With genetic engineering strategies and nanotechnology development, targeting CSCs using new technologies holds promise for initiating effective therapies. For instance, genetic engineering technology has been used to modify human neural stem cells to express interferon beta and cytosine deaminase. In vivo therapeutic experiments showed a significant increase in granzyme B levels within tumours and strengthened the cytotoxic activity of CD8^+^ T cells, thereby augmenting the anti‐tumour effect.^250^ In the inflammatory microenvironment, the splicing of ADAR1p150 exacerbates the generation of various CSCs and induces drug resistance. Rebecsinib, a small‐molecule inhibitor of ADAR1p150, can limit the proliferation and survival of leukaemia stem cells induced by the malignant TME.^251^ Additionally, targeted clearance of laryngeal CSCs is considered an effective strategy for cancer therapy. Previous studies have extensively proved that inhibiting the BMI‐1 gene can reduce the expansion and differentiation of laryngeal CSCs and enhance the chemotherapy efficacy of paclitaxel and other drugs. Researchers have designed nanoparticles (mPEG‐CS‐cRGD/BMI‐1RNAi‐PTX) with sustained‐release functionality, containing BMI‐1 siRNA and paclitaxel. These nanoparticles have low cytotoxicity and good anti‐tumour activity.^99^

## 8 CONCLUSION AND PROSPECTS

CSCs are a core driver population in tumours, playing a key role in tumourigenesis, metastasis, recurrence and treatment resistance through their self‐renewal, multidirectional differentiation and tumour‐initiating capabilities. In this paper, we systematically review the biological properties, markers and molecular regulatory mechanisms of CSCs, as well as their complex interactions with the TME. We also discuss clinical strategies for targeting CSCs.

The preservation of stem‐like characteristics in CSCs relies on the altered activation of several conserved signalling pathways, including Wnt/β‐catenin, Notch and Hedgehog. These pathways facilitate metabolic reprogramming and enable immune evasion in CSCs through the regulation of transcription factors (such as SOX2, NANOG and OCT4) and epigenetic changes. In addition, CSCs interact with various cells (e.g., TAMs, pericytes, MDSCs) and microbiota in the TME, constructing an immunosuppressive ecological niche that supports their survival. For example, CSCs evade immune surveillance by inducing macrophage polarisation towards the M2 type through the secretion of IL‐33 and TGF‐β or by inhibiting anti‐tumour immune responses by binding PD‐L1 to T‐cell surface receptors.^14^

Regarding clinical translation, the heterogeneity and dynamic plasticity of CSCs pose significant challenges. Although conventional chemotherapy and radiotherapy can eliminate most tumour cells, they may enrich the CSC population, leading to recurrence. In recent years, strategies targeting CSCs have been gradually developed, including the inhibition of key signalling pathways (e.g., the Wnt inhibitor LGK974 and the Hedgehog inhibitor Glasdegib),^155^, ^156^ intervention in metabolic dependence (e.g., ALDH1 inhibitors) and immune‐combination therapy (e.g., PD‐1/PD‐L1 inhibitors in combination with DC vaccines).^242^ Furthermore, single‐cell sequencing and spatial transcriptomics technologies have revealed the molecular heterogeneity of CSCs, providing new insights for precise classification. For instance, CD24^+^CD44^+^EpCAM^+^ gastric CSCs are significantly associated with poor patient prognosis, and the NNMT^+^/AQP5^+^ phenotype could serve as a potential diagnostic marker for gastric cardia cancer.^88^

Despite significant progress in CSC research, several bottlenecks remain in the clinical translation of these findings. 1. Heterogeneity and plasticity: CSCs are not a single, static population. The microenvironment dynamically regulates their phenotype and function. Future research should combine single‐cell multi‐omics technology to analyse the subpopulation characteristics of CSCs under different tumour types and therapeutic pressures and develop intervention strategies targeting plasticity regulatory nodes (e.g., EMT‐related pathways). 2. Temporal and spatial complexity of microenvironmental interactions: The interactions between CSCs and TMEs are spatiotemporally dependent. Simulation of the three‐dimensional dynamics of the tumour ecosystem using organoid models and in vivo imaging will help reveal the mechanisms by which CSCs maintain their ecological niche.^256^ For example, targeting methionine secreted by pericytes or microbiota metabolites may weaken the stemness of CSCs.^13^ 3. Therapeutic toxicity: targeting stemness pathways (e.g., the Notch inhibitor DAPT) may harm adult stem cells in tissues and organs^139^ (e.g., intestinal crypt stem cells), thereby limiting the clinical dosage. 4. Technological limitations: Traditional sequencing technologies cannot resolve the spatiotemporal heterogeneity of CSCs, while animal models (e.g., PDX) struggle to fully reproduce the immune and metabolic characteristics of the human TME.

To overcome the above challenges, future research should focus on the following directions: 1. Multi‐targeted combination therapy: single‐targeted strategies are prone to failure due to compensatory activation of signalling pathways. Future research should explore the synergistic effects of Wnt/Notch dual pathway inhibitors, epigenetic drugs (e.g., the EZH2 inhibitor Tazemetostat) and ICB.^158^ In addition, nanocarrier delivery systems (e.g., liposomes loaded with BMI‐1 siRNA) may enhance the drug‐specific killing of CSCs.^99^ 2. Immunometabolic regulation: CSCs maintain an immunosuppressive microenvironment through metabolic remodelling (e.g., enhanced glycolysis and lipid oxidation). Targeting metabolic enzymes (e.g., PCK2, ACSL4)^218^ or metabolites (e.g., lactate)^257^ may reverse T cell depletion. Modulating mitochondrial function (e.g., ISGylation‐mediated metabolic plasticity) or iron death sensitivity is expected to overcome chemoresistance.^228^ 3. New technology‐driven research innovations: CRISPR screening and cellular tracing technologies can systematically identify key genes dependent on CSCs. Spatial transcriptomics can elucidate the spatial distribution of CSCs in tumour tissues and their communication networks with neighbouring cells.^234^, ^258^ 4. Artificial intelligence‐assisted bioinformatics tools will accelerate the discovery of targets and drug design.

In summary, CSC research is transitioning from basic mechanism exploration to clinical translation by integrating multidisciplinary technologies, analysing the complexity of the tumour ecosystem and developing innovative therapeutic strategies. As research deepens, future therapeutic approaches will shift from a unidimensional ‘eradication of CSCs’ model towards a systematic ‘remodelling of the TME’. This strategic shift not only enhances our understanding of cancer biology but, more importantly, provides theoretical foundations for overcoming tumour drug resistance and improving clinical prognosis in patients.

## AUTHOR CONTRIBUTIONS

Huiling Wang and Junshu Li are co‐first authors. Huiling Wang designed the article and organised the data, Huiling Wang and Junshu Li wrote the manuscript, Junshu Li drew the figures, Fei Du checked the manuscript and Hongxin Deng verified the article data. All the authors read and approved the final manuscript.

## CONFLICT OF INTEREST STATEMENT

The authors declare no conflicts of interest.

## ETHICS STATEMENT

Not applicable.

## Data Availability

The data that support the findings of this study are available on request from the corresponding author. The data are not publicly available due to privacy or ethical restrictions.

## References

[1] Hanahan D . Hallmarks of cancer: new dimensions. Cancer Discov. 2022;12(1):31‐46. doi:10.1158/2159-8290.CD-21-1059 35022204

[2] Siegel RL , Kratzer TB , Giaquinto AN , Sung H , Jemal A . Cancer statistics, 2025. CA Cancer J Clin. 2025;75(1):10‐45. doi:10.3322/caac.21871 39817679 PMC11745215

[3] Bonnet D , Dick JE . Human acute myeloid leukemia is organized as a hierarchy that originates from a primitive hematopoietic cell. Nat Med. 1997;3(7):730‐737. doi:10.1038/nm0797-730 9212098

[4] Perez‐Gonzalez A , Bevant K , Blanpain C . Cancer cell plasticity during tumor progression, metastasis and response to therapy. Nat Cancer. 2023;4(8):1063‐1082. doi:10.1038/s43018-023-00595-y 37537300 PMC7615147

[5] Fanelli GN , Naccarato AG , Scatena C . Recent advances in cancer plasticity: cellular mechanisms, surveillance strategies, and therapeutic optimization. Front Oncol. 2020;10:569. doi:10.3389/fonc.2020.00569 32391266 PMC7188928

[6] Kreso A , Dick JE . Evolution of the cancer stem cell model. Cell Stem Cell. 2014;14(3):275‐291. doi:10.1016/j.stem.2014.02.006 24607403

[7] Duan H , Liu Y , Gao Z , Huang W . Recent advances in drug delivery systems for targeting cancer stem cells. Acta Pharm Sin B. 2021;11(1):55‐70. doi:10.1016/j.apsb.2020.09.016 33532180 PMC7838023

[8] Sarkar H , Lee E , Lopez‐Darwin SL , Kang Y . Deciphering normal and cancer stem cell niches by spatial transcriptomics: opportunities and challenges. Genes Dev. 2025;39(1‐2):64‐85. doi:10.1101/gad.351956.124 39496456 PMC11789490

[9] Glinsky GV . Stemness‘’ genomics law governs clinical behavior of human cancer: implications for decision making in disease management. J Clin Oncol. 2008;26(17):2846‐2853. doi:10.1200/JCO.2008.17.0266 18539963

[10] Chu X , Tian W , Ning J , et al. Cancer stem cells: advances in knowledge and implications for cancer therapy. Signal Transduct Target Ther. 2024;9(1):170. doi:10.1038/s41392-024-01851-y 38965243 PMC11224386

[11] de Visser KE , Joyce JA . The evolving tumor microenvironment: from cancer initiation to metastatic outgrowth. Cancer Cell. 2023;41(3):374‐403. doi:10.1016/j.ccell.2023.02.016 36917948

[12] Nusblat LM , Carroll MJ , Roth CM . Crosstalk between M2 macrophages and glioma stem cells. Cell Oncol (Dordr). 2017;40(5):471‐482. doi:10.1007/s13402-017-0337-5 28643230 PMC13001547

[13] Zhang C , Du Z , Gao Y , et al. Methionine secreted by tumor‐associated pericytes supports cancer stem cells in clear cell renal carcinoma. Cell Metab. 2024;36(4):778‐792. e10. 38378000 10.1016/j.cmet.2024.01.018

[14] Erickson HL , Taniguchi S , Raman A , Leitenberger JJ , Malhotra SV , Oshimori N . Cancer stem cells release interleukin‐33 within large oncosomes to promote immunosuppressive differentiation of macrophage precursors. Immunity. 2024;57(8):1908‐1922. e6. 39079535 10.1016/j.immuni.2024.07.004 PMC11324407

[15] Li YR , Fang Y , Lyu Z , Zhu Y , Yang L . Exploring the dynamic interplay between cancer stem cells and the tumor microenvironment: implications for novel therapeutic strategies. J Transl Med. 2023;21(1):686. doi:10.1186/s12967-023-04575-9 37784157 PMC10546755

[16] Sun S , Yang Q , Jiang D , Zhang Y . Nanobiotechnology augmented cancer stem cell guided management of cancer: liquid‐biopsy, imaging, and treatment. J Nanobiotechnol. 2024;22(1):176. doi:10.1186/s12951-024-02432-5 PMC11015566 38609981

[17] Yi W , Zhang J , Huang Y , et al. Ferritin‐mediated mitochondrial iron homeostasis is essential for the survival of hematopoietic stem cells and leukemic stem cells. Leukemia. 2024;38(5):1003‐1018. doi:10.1038/s41375-024-02169-y 38402368

[18] McCulloch EA , Till JE . Blast cells in acute myeloblastic leukemia: a model. Blood Cells. 1981;7(1):63‐77. 6964817

[19] Gisina A , Kholodenko I , Kim Y , Abakumov M , Lupatov A , Yarygin K . Glioma stem cells: novel data obtained by single‐cell sequencing. Int J Mol Sci. 2022;23(22):14224. doi:10.3390/ijms232214224 36430704 PMC9694247

[20] Shimokawa M , Ohta Y , Nishikori S , et al. Visualization and targeting of LGR5(+) human colon cancer stem cells. Nature. 2017;545(7653):187‐192. doi:10.1038/nature22081 28355176

[21] Sottoriva A , Verhoeff JJ , Borovski T , et al. Cancer stem cell tumor model reveals invasive morphology and increased phenotypical heterogeneity. Cancer Res. 2010;70(1):46‐56. doi:10.1158/0008-5472.CAN-09-3663 20048071

[22] Prager BC , Xie Q , Bao S , Rich JN . Cancer stem cells: the architects of the tumor ecosystem. Cell Stem Cell. 2019;24(1):41‐53. doi:10.1016/j.stem.2018.12.009 30609398 PMC6350931

[23] Jiang L , Hao Y , Shao C , et al. ADAR1‐mediated RNA editing links ganglioside catabolism to glioblastoma stem cell maintenance. J Clin Invest. 2022;132(6):e143397. doi:10.1172/JCI143397 35133980 PMC8920333

[24] Luo J , Gong L , Yang Y , et al. Enhanced mitophagy driven by ADAR1‐GLI1 editing supports the self‐renewal of cancer stem cells in HCC. Hepatology. 2024;79(1):61‐78. doi:10.1097/HEP.0000000000000299 36683360

[25] Cui J , Christin JR , Reisz JA , et al. Targeting ABCA12‐controlled ceramide homeostasis inhibits breast cancer stem cell function and chemoresistance. Sci Adv. 2023;9(48):eadh1891. doi:10.1126/sciadv.adh1891 38039374 PMC10691781

[26] Ravindran Menon D , Luo Y , Arcaroli JJ , et al. CDK1 interacts with Sox2 and promotes tumor initiation in human melanoma. Cancer Res. 2018;78(23):6561‐6574. doi:10.1158/0008-5472.CAN-18-0330 30297536 PMC6279496

[27] Li M , Zhong Y , Wang M . Fat1 suppresses the tumor‐initiating ability of nonsmall cell lung cancer cells by promoting Yes‐associated protein 1 nuclear‐cytoplasmic translocation. Environ Toxicol. 2021;36(11):2333‐2341. doi:10.1002/tox.23347 34390292

[28] Barker N , van Es JH , Kuipers J , et al. Identification of stem cells in small intestine and colon by marker gene Lgr5. Nature. 2007;449(7165):1003‐1007. doi:10.1038/nature06196 17934449

[29] Easwaran H , Tsai HC , Baylin SB . Cancer epigenetics: tumor heterogeneity, plasticity of stem‐like states, and drug resistance. Mol Cell. 2014;54(5):716‐727. doi:10.1016/j.molcel.2014.05.015 24905005 PMC4103691

[30] Nowell PC . The clonal evolution of tumor cell populations. Science. 1976;194(4260):23‐28. doi:10.1126/science.959840 959840

[31] Teixeira MR , Heim S . Cytogenetic analysis of tumor clonality. Adv Cancer Res. 2011;112:127‐149. doi:10.1016/B978-0-12-387688-1.00005-3 21925303

[32] Snippert HJ , van der Flier LG , Sato T , et al. Intestinal crypt homeostasis results from neutral competition between symmetrically dividing Lgr5 stem cells. Cell. 2010;143(1):134‐144. doi:10.1016/j.cell.2010.09.016 20887898

[33] Lopez‐Garcia C , Klein AM , Simons BD , Winton DJ . Intestinal stem cell replacement follows a pattern of neutral drift. Science. 2010;330(6005):822‐825. doi:10.1126/science.1196236 20929733

[34] Leushacke M , Ng A , Galle J , Loeffler M , Barker N . Lgr5(+) gastric stem cells divide symmetrically to effect epithelial homeostasis in the pylorus. Cell Rep. 2013;5(2):349‐356. doi:10.1016/j.celrep.2013.09.025 24209744

[35] Doupe DP , Klein AM , Simons BD , Jones PH . The ordered architecture of murine ear epidermis is maintained by progenitor cells with random fate. Dev Cell. 2010;18(2):317‐323. doi:10.1016/j.devcel.2009.12.016 20159601

[36] Xue M , Dong L , Zhang H , et al. METTL16 promotes liver cancer stem cell self‐renewal via controlling ribosome biogenesis and mRNA translation. J Hematol Oncol. 2024;17(1):7. doi:10.1186/s13045-024-01526-9 38302992 PMC10835888

[37] Han L , Dong L , Leung K , et al. METTL16 drives leukemogenesis and leukemia stem cell self‐renewal by reprogramming BCAA metabolism. Cell Stem Cell. 2023;30(1):52‐68. doi:10.1016/j.stem.2022.12.006. e13. 36608679 PMC9838187

[38] Wang X , Chen Y , Wang X , et al. Stem cell factor SOX2 confers ferroptosis resistance in lung cancer via upregulation of SLC7A11. Cancer Res. 2021;81(20):5217‐5229. doi:10.1158/0008-5472.CAN-21-0567 34385181 PMC8530936

[39] Marjanovic ND , Weinberg RA , Chaffer CL . Cell plasticity and heterogeneity in cancer. Clin Chem. 2013;59(1):168‐179. doi:10.1373/clinchem.2012.184655 23220226 PMC6220421

[40] Cabrera MC , Hollingsworth RE , Hurt EM . Cancer stem cell plasticity and tumor hierarchy. World J Stem Cells. 2015;7(1):27‐36. doi:10.4252/wjsc.v7.i1.27 25621103 PMC4300934

[41] Cirri P , Chiarugi P . Cancer‐associated‐fibroblasts and tumour cells: a diabolic liaison driving cancer progression. Cancer Metastasis Rev. 2012;31(1–2):195‐208. doi:10.1007/s10555-011-9340-x 22101652

[42] Chan JM , Zaidi S , Love JR , et al. Lineage plasticity in prostate cancer depends on JAK/STAT inflammatory signaling. Science. 2022;377(6611):1180‐1191. doi:10.1126/science.abn0478 35981096 PMC9653178

[43] Mauri F , Schepkens C , Lapouge G , et al. NR2F2 controls malignant squamous cell carcinoma state by promoting stemness and invasion and repressing differentiation. Nat Cancer. 2021;2(11):1152‐1169. doi:10.1038/s43018-021-00287-5 35122061 PMC7615150

[44] Zhao R , Guo X , Zhang G , et al. CMYC‐initiated HNF1A‐AS1 overexpression maintains the stemness of gastric cancer cells. Cell Death Dis. 2024;15(4):288. doi:10.1038/s41419-024-06673-y 38654006 PMC11039746

[45] Shahzad U , Nikolopoulos M , Li C , et al. CASCADES, a novel SOX2 super‐enhancer‐associated long noncoding RNA, regulates cancer stem cell specification and differentiation in glioblastoma. Mol Oncol. 2025;19(3):764‐784. doi:10.1002/1878-0261.13735 39323013 PMC11887672

[46] Chen Z , He Q , Lu T , et al. mcPGK1‐dependent mitochondrial import of PGK1 promotes metabolic reprogramming and self‐renewal of liver TICs. Nat Commun. 2023;14(1):1121. doi:10.1038/s41467-023-36651-5 36849569 PMC9971191

[47] Saw PE , Liu Q , Wong PP , Song E . Cancer stem cell mimicry for immune evasion and therapeutic resistance. Cell Stem Cell. 2024;31(8):1101‐1112. doi:10.1016/j.stem.2024.06.003 38925125

[48] Li H , Li X , Liu S , et al. Programmed cell death‐1 (PD‐1) checkpoint blockade in combination with a mammalian target of rapamycin inhibitor restrains hepatocellular carcinoma growth induced by hepatoma cell‐intrinsic PD‐1. Hepatology. 2017;66(6):1920‐1933. doi:10.1002/hep.29360 28732118

[49] Wang X , Yang X , Zhang C , et al. Tumor cell‐intrinsic PD‐1 receptor is a tumor suppressor and mediates resistance to PD‐1 blockade therapy. Proc Natl Acad Sci U S A. 2020;117(12):6640‐6650. doi:10.1073/pnas.1921445117 32161124 PMC7104341

[50] Sun H , Yao N , Cheng S , et al. Cancer stem‐like cells directly participate in vasculogenic mimicry channels in triple‐negative breast cancer. Cancer Biol Med. 2019;16(2):299‐311. doi:10.20892/j.issn.2095-3941.2018.0209 31516750 PMC6713644

[51] Ricci‐Vitiani L , Pallini R , Biffoni M , et al. Tumour vascularization via endothelial differentiation of glioblastoma stem‐like cells. Nature. 2010;468(7325):824‐828. doi:10.1038/nature09557 21102434

[52] Wilkinson AL , Zorzan I , Rugg‐Gunn PJ . Epigenetic regulation of early human embryo development. Cell Stem Cell. 2023;30(12):1569‐1584. doi:10.1016/j.stem.2023.09.010 37858333

[53] Loh JJ , Ma S . Hallmarks of cancer stemness. Cell Stem Cell. 2024;31(5):617‐639. doi:10.1016/j.stem.2024.04.004 38701757

[54] Gupta PB , Fillmore CM , Jiang G , et al. Stochastic state transitions give rise to phenotypic equilibrium in populations of cancer cells. Cell. 2011;146(4):633‐644. doi:10.1016/j.cell.2011.07.026 21854987

[55] de Sousa e Melo F , Kurtova AV , Harnoss JM , et al. A distinct role for Lgr5(+) stem cells in primary and metastatic colon cancer. Nature. 2017;543(7647):676‐680. doi:10.1038/nature21713 28358093

[56] Chen J , Li Y , Yu TS , et al. A restricted cell population propagates glioblastoma growth after chemotherapy. Nature. 2012;488(7412):522‐526. doi:10.1038/nature11287 22854781 PMC3427400

[57] Suva ML , Rheinbay E , Gillespie SM , et al. Reconstructing and reprogramming the tumor‐propagating potential of glioblastoma stem‐like cells. Cell. 2014;157(3):580‐594. doi:10.1016/j.cell.2014.02.030 24726434 PMC4004670

[58] Ohta Y , Fujii M , Takahashi S , et al. Cell‐matrix interface regulates dormancy in human colon cancer stem cells. Nature. 2022;608(7924):784‐794. doi:10.1038/s41586-022-05043-y 35798028

[59] Ding XW , Wu JH , Jiang CP . ABCG2: a potential marker of stem cells and novel target in stem cell and cancer therapy. Life Sci. 2010;86(17–18):631‐637. doi:10.1016/j.lfs.2010.02.012 20159023

[60] Wang Z , Yip LY , Lee JHJ , et al. Methionine is a metabolic dependency of tumor‐initiating cells. Nat Med. 2019;25(5):825‐837. doi:10.1038/s41591-019-0423-5 31061538

[61] Kozono D , Li J , Nitta M , et al. Dynamic epigenetic regulation of glioblastoma tumorigenicity through LSD1 modulation of MYC expression. Proc Natl Acad Sci U S A. 2015;112(30):E4055‐64. doi:10.1073/pnas.1501967112 26159421 PMC4522819

[62] Xu C , Jin G , Wu H , et al. SIRPgamma‐expressing cancer stem‐like cells promote immune escape of lung cancer via Hippo signaling. J Clin Invest. 2022;132(5):e141797. doi:10.1172/JCI141797 35229723 PMC8884909

[63] Pérez‐Núñez I , Rozalen C , Palomeque JÁ , et al. LCOR mediates interferon‐independent tumor immunogenicity and responsiveness to immune‐checkpoint blockade in triple‐negative breast cancer. Nat Cancer. 2022;3(3):355‐370. 35301507 10.1038/s43018-022-00339-4

[64] Miao Y , Yang H , Levorse J , et al. Adaptive immune resistance emerges from tumor‐initiating stem cells. Cell. 2019;177(5):1172‐1186. doi:10.1016/j.cell.2019.03.025. e14. 31031009 PMC6525024

[65] Wang G , Lu X , Dey P , et al. Targeting YAP‐dependent MDSC infiltration impairs tumor progression. Cancer Discov. 2016;6(1):80‐95. doi:10.1158/2159-8290.CD-15-0224 26701088 PMC4707102

[66] Bo Y , Zhou J , Cai K , et al. Leveraging intracellular ALDH1A1 activity for selective cancer stem‐like cell labeling and targeted treatment via in vivo click reaction. Proc Natl Acad Sci U S A. 2023;120(36):e2302342120. doi:10.1073/pnas.2302342120 37639589 PMC10483628

[67] Zhai Y , Li G , Pan C , et al. The development and potent antitumor efficacy of CD44/CD133 dual‐targeting IL7Ralpha‐armored CAR‐T cells against glioblastoma. Cancer Lett. 2025;614:217541. doi:10.1016/j.canlet.2025.217541 39952598

[68] Sloan AR , Thapliyal M , Lathia JD . New T‐cell therapies for brain metastasis, CD133 in the driver's seat. Clin Cancer Res. 2024;30(3):477‐479. doi:10.1158/1078-0432.CCR-23-3051 38038689 PMC10842869

[69] Yang C , You J , Pan Q , et al. Targeted delivery of a PD‐1‐blocking scFv by CD133‐specific CAR‐T cells using nonviral sleeping beauty transposition shows enhanced antitumour efficacy for advanced hepatocellular carcinoma. BMC Med. 2023;21(1):327. doi:10.1186/s12916-023-03016-0 37635247 PMC10464109

[70] Ouhtit A , Rizeq B , Saleh HA , Rahman MM , Zayed H . Novel CD44‐downstream signaling pathways mediating breast tumor invasion. Int J Biol Sci. 2018;14(13):1782‐1790. doi:10.7150/ijbs.23586 30443182 PMC6231220

[71] Al‐Hajj M , Wicha MS , Benito‐Hernandez A , Morrison SJ , Clarke MF . Prospective identification of tumorigenic breast cancer cells. Proc Natl Acad Sci U S A. 2003;100(7):3983‐3988. doi:10.1073/pnas.0530291100 12629218 PMC153034

[72] Liu X , Ye Y , Zhu L , et al. Niche stiffness sustains cancer stemness via TAZ and NANOG phase separation. Nat Commun. 2023;14(1):238. doi:10.1038/s41467-023-35856-y 36646707 PMC9842735

[73] Zeuner A , Todaro M , Stassi G , De Maria R . Colorectal cancer stem cells: from the crypt to the clinic. Cell Stem Cell. 2014;15(6):692‐705. doi:10.1016/j.stem.2014.11.012 25479747

[74] Huang Q , Liu L , Xiao D , et al. CD44+ lung cancer stem cell‐derived pericyte‐like cells cause brain metastases through GPR124‐enhanced trans‐endothelial migration. Cancer Cell. 2023;41(9):1621‐1636. e8. 37595587 10.1016/j.ccell.2023.07.012

[75] Wang X , Cai J , Zhao L , et al. NUMB suppression by miR‐9‐5P enhances CD44+ prostate cancer stem cell growth and metastasis. Sci Rep. 2021;11(1):11210. 34045601 10.1038/s41598-021-90700-x PMC8160147

[76] Praharaj PP , Singh A , Patra S , Bhutia SK . Co‐targeting autophagy and NRF2 signaling triggers mitochondrial superoxide to sensitize oral cancer stem cells for cisplatin‐induced apoptosis. Free Radic Biol Med. 2023;207:72‐88. 37423560 10.1016/j.freeradbiomed.2023.07.008

[77] Dall'antonia F , Pavkov‐Keller T , Zangger K , Keller W . Structure of allergens and structure based epitope predictions. Methods. 2014;66(1):3‐21. doi:10.1016/j.ymeth.2013.07.024 23891546 PMC3969231

[78] Yang L , Shi P , Zhao G , et al. Targeting cancer stem cell pathways for cancer therapy. Signal Transduct Target Ther. 2020;5(1):8. doi:10.1038/s41392-020-0110-5 32296030 PMC7005297

[79] Pang L , Dunterman M , Guo S , et al. Kunitz‐type protease inhibitor TFPI2 remodels stemness and immunosuppressive tumor microenvironment in glioblastoma. Nat Immunol. 2023;24(10):1654‐1670. doi:10.1038/s41590-023-01605-y 37667051 PMC10775912

[80] Zhou L , Yu KH , Wong TL , et al. Lineage tracing and single‐cell analysis reveal proliferative Prom1+ tumour‐propagating cells and their dynamic cellular transition during liver cancer progression. Gut. 2022;71(8):1656‐1668. doi:10.1136/gutjnl-2021-324321 34588223

[81] Wang R , Li Y , Tsung A , et al. iNOS promotes CD24(+)CD133(+) liver cancer stem cell phenotype through a TACE/ADAM17‐dependent Notch signaling pathway. Proc Natl Acad Sci U S A. 2018;115(43):E10127‐E10136. doi:10.1073/pnas.1722100115 30297396 PMC6205478

[82] Rodrigues A , Silva SLR , Dias I , et al. Piplartine eliminates CD34 + AML stem/progenitor cells by inducing oxidative stress and suppressing NF‐kappaB signalling. Cell Death Discov. 2024;10(1):147. doi:10.1038/s41420-024-01909-4 38503729 PMC10951277

[83] Kuepper MK , Butow M , Herrmann O , et al. Stem cell persistence in CML is mediated by extrinsically activated JAK1‐STAT3 signaling. Leukemia. 2019;33(8):1964‐1977. doi:10.1038/s41375-019-0427-7 30842608

[84] Debaugnies M , Rodriguez‐Acebes S , Blondeau J , et al. RHOJ controls EMT‐associated resistance to chemotherapy. Nature. 2023;616(7955):168‐175. doi:10.1038/s41586-023-05838-7 36949199 PMC10076223

[85] Ng KY , Shea QT , Wong TL , et al. Chemotherapy‐enriched THBS2‐deficient cancer stem cells drive hepatocarcinogenesis through matrix softness induced histone H3 modifications. Adv Sci (Weinh). 2021;8(5):2002483. doi:10.1002/advs.202002483 33717837 PMC7927606

[86] Park DJ , Sung PS , Kim J‐H , et al. EpCAM‐high liver cancer stem cells resist natural killer cell‐mediated cytotoxicity by upregulating CEACAM1. J Immunother Cancer. 2020;8(1):e000301. 32221015 10.1136/jitc-2019-000301 PMC7206970

[87] Gomez‐Gallegos AA , Ramirez‐Vidal L , Becerril‐Rico J , et al. CD24+CD44+CD54+EpCAM+ gastric cancer stem cells predict tumor progression and metastasis: clinical and experimental evidence. Stem Cell Res Ther. 2023;14(1):16. doi:10.1186/s13287-023-03241-7 36737794 PMC9898964

[88] Wang Z , Wang Q , Chen C , et al. NNMT enriches for AQP5(+) cancer stem cells to drive malignant progression in early gastric cardia adenocarcinoma. Gut. 2023;73(1):63‐77. doi:10.1136/gutjnl-2022-328408 36977555

[89] Inukai M , Hara A , Yasui Y , Kumabe T , Matsumoto T , Saegusa M . Hypoxia‐mediated cancer stem cells in pseudopalisades with activation of hypoxia‐inducible factor‐1alpha/Akt axis in glioblastoma. Hum Pathol. 2015;46(10):1496‐1505. doi:10.1016/j.humpath.2015.06.008 26256949

[90] Zhu Y , Huang S , Chen S , et al. SOX2 promotes chemoresistance, cancer stem cells properties, and epithelial‐mesenchymal transition by beta‐catenin and Beclin1/autophagy signaling in colorectal cancer. Cell Death Dis. 2021;12(5):449. doi:10.1038/s41419-021-03733-5 33953166 PMC8100126

[91] Lu Y , Zhu Y , Deng S , et al. Targeting the sonic Hedgehog pathway to suppress the expression of the cancer stem cell (CSC)‐related transcription factors and CSC‐driven thyroid tumor growth. Cancers (Basel). 2021;13(3):418. doi:10.3390/cancers13030418 33499351 PMC7866109

[92] Huang C , Lu H , Li J , et al. SOX2 regulates radioresistance in cervical cancer via the Hedgehog signaling pathway. Gynecol Oncol. 2018;151(3):533‐541. doi:10.1016/j.ygyno.2018.10.005 30336948

[93] Patil K , Khan FB , Akhtar S , Ahmad A , Uddin S . The plasticity of pancreatic cancer stem cells: implications in therapeutic resistance. Cancer Metastasis Rev. 2021;40(3):691‐720. doi:10.1007/s10555-021-09979-x 34453639 PMC8556195

[94] Woo SR , Lee HJ , Oh SJ , et al. Stabilization of HDAC1 via TCL1‐pAKT‐CHFR axis is a key element for NANOG‐mediated multi‐resistance and stem‐like phenotype in immune‐edited tumor cells. Biochem Biophys Res Commun. 2018;503(3):1812‐1818. doi:10.1016/j.bbrc.2018.07.118 30060952

[95] Yang W , Kim D , Kim DK , Choi KU , Suh DS , Kim JH . Therapeutic strategies for targeting ovarian cancer stem cells. Int J Mol Sci. 2021;22(10):5059. doi:10.3390/ijms22105059 34064635 PMC8151268

[96] Safa AR , Saadatzadeh MR , Cohen‐Gadol AA , Pollok KE , Bijangi‐Vishehsaraei K . Glioblastoma stem cells (GSCs) epigenetic plasticity and interconversion between differentiated non‐GSCs and GSCs. Genes Dis. 2015;2(2):152‐163. doi:10.1016/j.gendis.2015.02.001 26137500 PMC4484766

[97] Xu J , Li L , Shi P , Cui H , Yang L . The crucial roles of Bmi‐1 in cancer: implications in pathogenesis, metastasis, drug resistance, and targeted therapies. Int J Mol Sci. 2022;23(15):8231. doi:10.3390/ijms23158231 35897796 PMC9367737

[98] Nallasamy P , Nimmakayala RK , Parte S , Are AC , Batra SK , Ponnusamy MP . Tumor microenvironment enriches the stemness features: the architectural event of therapy resistance and metastasis. Mol Cancer. 2022;21(1):225. doi:10.1186/s12943-022-01682-x 36550571 PMC9773588

[99] Xu X , Zhou T , Wei X , Jiang X , Cao J . Application of mPEG‐CS‐cRGD/Bmi‐1RNAi‐PTX nanoparticles in suppression of laryngeal cancer by targeting cancer stem cells. Drug Deliv. 2023;30(1):2180112. 38095348 10.1080/10717544.2023.2180112 PMC9946312

[100] Sarkar P , Basu K , Sarkar P , et al. Correlations of aldehyde dehydrogenase‐1 (ALDH1) expression with traditional prognostic parameters and different molecular subtypes of breast carcinoma. Clujul Med. 2018;91(2):181‐187. doi:10.15386/cjmed-925 29785156 PMC5958983

[101] Liu C , Qiang J , Deng Q , et al. ALDH1A1 activity in tumor‐initiating cells remodels myeloid‐derived suppressor cells to promote breast cancer progression. Cancer Res. 2021;81(23):5919‐5934. doi:10.1158/0008-5472.CAN-21-1337 34580061

[102] Dalerba P , Dylla SJ , Park IK , et al. Phenotypic characterization of human colorectal cancer stem cells. Proc Natl Acad Sci U S A. 2007;104(24):10158‐10163. doi:10.1073/pnas.0703478104 17548814 PMC1891215

[103] Nieder J , Tomaschek F . Maltese as a merger of two worlds: a cross‐language approach to phonotactic classification. PLoS ONE. 2023;18(4):e0284534. doi:10.1371/journal.pone.0284534 37071659 PMC10112784

[104] Roudi R , Korourian A , Shariftabrizi A , Madjd Z . Differential expression of cancer stem cell markers ALDH1 and CD133 in various lung cancer subtypes. Cancer Invest. 2015;33(7):294‐302. doi:10.3109/07357907.2015.1034869 26046383

[105] Wilson BJ , Saab KR , Ma J , et al. ABCB5 maintains melanoma‐initiating cells through a proinflammatory cytokine signaling circuit. Cancer Res. 2014;74(15):4196‐4207. doi:10.1158/0008-5472.CAN-14-0582 24934811 PMC4119553

[106] Lee CAA , Banerjee P , Wilson BJ , et al. Targeting the ABC transporter ABCB5 sensitizes glioblastoma to temozolomide‐induced apoptosis through a cell‐cycle checkpoint regulation mechanism. J Biol Chem. 2020;295(22):7774‐7788. doi:10.1074/jbc.RA120.013778 32317280 PMC7261782

[107] Olive KP , Jacobetz MA , Davidson CJ , et al. Inhibition of Hedgehog signaling enhances delivery of chemotherapy in a mouse model of pancreatic cancer. Science. 2009;324(5933):1457‐1461. doi:10.1126/science.1171362 19460966 PMC2998180

[108] Kukal S , Guin D , Rawat C , et al. Multidrug efflux transporter ABCG2: expression and regulation. Cell Mol Life Sci. 2021;78(21–22):6887‐6939. doi:10.1007/s00018-021-03901-y 34586444 PMC11072723

[109] Shen M , Xu Z , Xu W , et al. Inhibition of ATM reverses EMT and decreases metastatic potential of cisplatin‐resistant lung cancer cells through JAK/STAT3/PD‐L1 pathway. J Exp Clin Cancer Res. 2019;38(1):149. doi:10.1186/s13046-019-1161-8 30961670 PMC6454747

[110] Alwosaibai K , Aalmri S , Mashhour M , et al. PD‐L1 is highly expressed in ovarian cancer and associated with cancer stem cells populations expressing CD44 and other stem cell markers. BMC Cancer. 2023;23(1):13. 36604635 10.1186/s12885-022-10404-x PMC9814309

[111] Zheng L , Lu J , Kong D , Zhan Y . Single‐cell sequencing analysis revealed that WDR72 was a novel cancer stem cells related gene in gastric cancer. Heliyon. 2024;10(15):e35549. 39170171 10.1016/j.heliyon.2024.e35549 PMC11336769

[112] Bhatia M , Wang JC , Kapp U , Bonnet D , Dick JE . Purification of primitive human hematopoietic cells capable of repopulating immune‐deficient mice. Proc Natl Acad Sci U S A. 1997;94(10):5320‐5325. doi:10.1073/pnas.94.10.5320 9144235 PMC24676

[113] Wang R , Chadalavada K , Wilshire J , et al. Glioblastoma stem‐like cells give rise to tumour endothelium. Nature. 2010;468(7325):829‐833. doi:10.1038/nature09624 21102433

[114] Walcher L , Kistenmacher AK , Suo H , et al. Cancer stem cells‐origins and biomarkers: perspectives for targeted personalized therapies. Front Immunol. 2020;11:1280. doi:10.3389/fimmu.2020.01280 32849491 PMC7426526

[115] Zhu Z , Mesci P , Bernatchez JA , et al. Zika virus targets glioblastoma stem cells through a SOX2‐integrin alpha(v)beta(5) axis. Cell Stem Cell. 2020;26(2):187‐204. doi:10.1016/j.stem.2019.11.016. e10. 31956038 PMC9628766

[116] Najafzadeh B , Asadzadeh Z , Motafakker Azad R , et al. The oncogenic potential of NANOG: an important cancer induction mediator. J Cell Physiol. 2021;236(4):2443‐2458. doi:10.1002/jcp.30063 32960465

[117] Grubelnik G , Bostjancic E , Pavlic A , Kos M , Zidar N . NANOG expression in human development and cancerogenesis. Exp Biol Med (Maywood). 2020;245(5):456‐464. doi:10.1177/1535370220905560 32041418 PMC7082888

[118] Charafe‐Jauffret E , Ginestier C , Iovino F , et al. Aldehyde dehydrogenase 1‐positive cancer stem cells mediate metastasis and poor clinical outcome in inflammatory breast cancer. Clin Cancer Res. 2010;16(1):45‐55. doi:10.1158/1078-0432.CCR-09-1630 20028757 PMC2874875

[119] Zanoni M , Bravaccini S , Fabbri F , Arienti C . Emerging roles of aldehyde dehydrogenase isoforms in anti‐cancer therapy resistance. Front Med (Lausanne). 2022;9:795762. doi:10.3389/fmed.2022.795762 35299840 PMC8920988

[120] Li T , Su Y , Mei Y , et al. ALDH1A1 is a marker for malignant prostate stem cells and predictor of prostate cancer patients' outcome. Lab Invest. 2010;90(2):234‐244. doi:10.1038/labinvest.2009.127 20010854 PMC3552330

[121] Duan JJ , Wang D , Cai J , et al. An aldehyde dehydrogenase 1A3 inhibitor attenuates the metastasis of human colorectal cancer. Cancer Lett. 2022;536:215662. doi:10.1016/j.canlet.2022.215662 35331786

[122] Tian S , Liu DH , Wang D , Ren F , Xia P . Aldehyde dehydrogenase 1 (ALDH1) promotes the toxicity of TRAIL in non‐small cell lung cancer cells via post‐transcriptional regulation of MEK‐1 expression. Cell Physiol Biochem. 2018;51(1):217‐227. doi:10.1159/000495202 30448845

[123] Allikmets R , Schriml LM , Hutchinson A , Romano‐Spica V , Dean M . A human placenta‐specific ATP‐binding cassette gene (ABCP) on chromosome 4q22 that is involved in multidrug resistance. Cancer Res. 1998;58(23):5337‐5339. 9850061

[124] Vahidian F , Duijf PHG , Safarzadeh E , Derakhshani A , Baghbanzadeh A , Baradaran B . Interactions between cancer stem cells, immune system and some environmental components: friends or foes?. Immunol Lett. 2019;208:19‐29. doi:10.1016/j.imlet.2019.03.004 30862442

[125] Aponte PM , Caicedo A . Stemness in cancer: stem cells, cancer stem cells, and their microenvironment. Stem Cells Int. 2017;2017:5619472. doi:10.1155/2017/5619472 28473858 PMC5394399

[126] Brugnoli F , Grassilli S , Al‐Qassab Y , Capitani S , Bertagnolo V . CD133 in breast cancer cells: more than a stem cell marker. J Oncol. 2019;2019:7512632. doi:10.1155/2019/7512632 31636668 PMC6766124

[127] Kozovska Z , Patsalias A , Bajzik V , et al. ALDH1A inhibition sensitizes colon cancer cells to chemotherapy. BMC Cancer. 2018;18(1):656. doi:10.1186/s12885-018-4572-6 29902974 PMC6003038

[128] Liu J , Xiao Q , Xiao J , et al. Wnt/beta‐catenin signalling: function, biological mechanisms, and therapeutic opportunities. Signal Transduct Target Ther. 2022;7(1):3. doi:10.1038/s41392-021-00762-6 34980884 PMC8724284

[129] Song P , Gao Z , Bao Y , et al. Wnt/beta‐catenin signaling pathway in carcinogenesis and cancer therapy. J Hematol Oncol. 2024;17(1):46. doi:10.1186/s13045-024-01563-4 38886806 PMC11184729

[130] Daniels DL , Weis WI . Beta‐catenin directly displaces Groucho/TLE repressors from TCF/LEF in Wnt‐mediated transcription activation. Nat Struct Mol Biol. 2005;12(4):364‐371. doi:10.1038/nsmb912 15768032

[131] Ma ZQ , Feng YT , Guo K , et al. Melatonin inhibits ESCC tumor growth by mitigating the HDAC7/beta‐catenin/c‐Myc positive feedback loop and suppressing the USP10‐maintained HDAC7 protein stability. Mil Med Res. 2022;9(1):54. doi:10.1186/s40779-022-00412-0 36163081 PMC9513894

[132] Yu J , Liu D , Sun X , et al. CDX2 inhibits the proliferation and tumor formation of colon cancer cells by suppressing Wnt/beta‐catenin signaling via transactivation of GSK‐3beta and Axin2 expression. Cell Death Dis. 2019;10(1):26. doi:10.1038/s41419-018-1263-9 30631044 PMC6328578

[133] Chen Y , Zhao H , Liang W , et al. Autophagy regulates the cancer stem cell phenotype of head and neck squamous cell carcinoma through the noncanonical FOXO3/SOX2 axis. Oncogene. 2022;41(5):634‐646. 34795388 10.1038/s41388-021-02115-7 PMC8799462

[134] Huang Y , Sheng H , Xiao Y , et al. Wnt/beta‐catenin inhibitor ICG‐001 enhances the antitumor efficacy of radiotherapy by increasing radiation‐induced DNA damage and improving tumor immune microenvironment in hepatocellular carcinoma. Radiother Oncol. 2021;162:34‐44. doi:10.1016/j.radonc.2021.06.034 34214613

[135] Ji L , Qian W , Gui L , et al. Blockade of beta‐catenin‐induced CCL28 suppresses gastric cancer progression via inhibition of Treg cell infiltration. Cancer Res. 2020;80(10):2004‐2016. doi:10.1158/0008-5472.CAN-19-3074 32156780

[136] Bray SJ . Notch signalling: a simple pathway becomes complex. Nat Rev Mol Cell Biol. 2006;7(9):678‐689. doi:10.1038/nrm2009 16921404

[137] Wang Y , Wang Y , Chen H , Liang Q . Endothelial cells promote formation of medulloblastoma stem‐like cells via Notch pathway activation. J Mol Neurosci. 2017;63(2):152‐158. doi:10.1007/s12031-017-0965-2 28856557

[138] Choi S , Yu J , Park A , et al. BMP‐4 enhances epithelial mesenchymal transition and cancer stem cell properties of breast cancer cells via Notch signaling. Sci Rep. 2019;9(1):11724. doi:10.1038/s41598-019-48190-5 31409851 PMC6692307

[139] Pindiprolu S , Krishnamurthy PT , Dev C , Chintamaneni PK . DR5 antibody conjugated lipid‐based nanocarriers of gamma‐secretase inhibitor for the treatment of triple negative breast cancer. Chem Phys Lipids. 2021;235:105033. doi:10.1016/j.chemphyslip.2020.105033 33385372

[140] Lopez‐Guerra M , Xargay‐Torrent S , Fuentes P , et al. Specific NOTCH1 antibody targets DLL4‐induced proliferation, migration, and angiogenesis in NOTCH1‐mutated CLL cells. Oncogene. 2020;39(6):1185‐1197. doi:10.1038/s41388-019-1053-6 31616059 PMC7002297

[141] Zhang Y , Beachy PA . Cellular and molecular mechanisms of Hedgehog signalling. Nat Rev Mol Cell Biol. 2023;24(9):668‐687. doi:10.1038/s41580-023-00591-1 36932157 PMC12140928

[142] Johnson RL , Rothman AL , Xie J , et al. Human homolog of patched, a candidate gene for the basal cell nevus syndrome. Science. 1996;272(5268):1668‐1671. doi:10.1126/science.272.5268.1668 8658145

[143] Lee JJ , Perera RM , Wang H , et al. Stromal response to Hedgehog signaling restrains pancreatic cancer progression. Proc Natl Acad Sci U S A. 2014;111(30):E3091‐100. doi:10.1073/pnas.1411679111 25024225 PMC4121834

[144] Teglund S , Toftgard R . Hedgehog beyond medulloblastoma and basal cell carcinoma. Biochim Biophys Acta. 2010;1805(2):181‐208. doi:10.1016/j.bbcan.2010.01.003 20085802

[145] Jiang J . Hedgehog signaling mechanism and role in cancer. Semin Cancer Biol. 2022;85:107‐122. doi:10.1016/j.semcancer.2021.04.003 33836254 PMC8492792

[146] Catenacci DV , Junttila MR , Karrison T , et al. Randomized phase Ib/II study of gemcitabine plus placebo or vismodegib, a Hedgehog pathway inhibitor, in patients with metastatic pancreatic cancer. J Clin Oncol. 2015;33(36):4284‐4292. doi:10.1200/JCO.2015.62.8719 26527777 PMC4678179

[147] Steele NG , Biffi G , Kemp SB , et al. Inhibition of Hedgehog signaling alters fibroblast composition in pancreatic cancer. Clin Cancer Res. 2021;27(7):2023‐2037. doi:10.1158/1078-0432.CCR-20-3715 33495315 PMC8026631

[148] Wang Q , Liang N , Yang T , et al. DNMT1‐mediated methylation of BEX1 regulates stemness and tumorigenicity in liver cancer. J Hepatol. 2021;75(5):1142‐1153. doi:10.1016/j.jhep.2021.06.025 34217777

[149] Debeb BG , Lacerda L , Xu W , et al. Histone deacetylase inhibitors stimulate dedifferentiation of human breast cancer cells through WNT/beta‐catenin signaling. Stem Cells. 2012;30(11):2366‐2377. doi:10.1002/stem.1219 22961641 PMC4545658

[150] Shamsian A , Sepand MR , Javaheri Kachousangi M , et al. Targeting tumorigenicity of breast cancer stem cells using SAHA/Wnt‐b catenin antagonist loaded onto protein corona of gold nanoparticles. Int J Nanomed. 2020;15:4063‐4078. doi:10.2147/IJN.S234636 PMC7295335 32606664

[151] Lin Y , Jian Z , Jin H , et al. Long non‐coding RNA DLGAP1‐AS1 facilitates tumorigenesis and epithelial‐mesenchymal transition in hepatocellular carcinoma via the feedback loop of miR‐26a/b‐5p/IL‐6/JAK2/STAT3 and Wnt/beta‐catenin pathway. Cell Death Dis. 2020;11(1):34. doi:10.1038/s41419-019-2188-7 31949128 PMC6965175

[152] Kaltschmidt C , Banz‐Jansen C , Benhidjeb T , et al. A role for NF‐kappaB in organ specific cancer and cancer stem cells. Cancers (Basel). 2019;11(5):655. doi:10.3390/cancers11050655 31083587 PMC6563002

[153] Kim DA , Choi HS , Ryu ES , et al. Tannic acid attenuates the formation of cancer stem cells by inhibiting NF‐kappaB‐mediated phenotype transition of breast cancer cells. Am J Cancer Res. 2019;9(8):1664‐1681. 31497349 PMC6726983

[154] Kaltschmidt B , Witte KE , Greiner JFW , Weissinger F , Kaltschmidt C . Targeting NF‐kappaB signaling in cancer stem cells: a narrative review. Biomedicines. 2022;10(2):261. doi:10.3390/biomedicines10020261 35203471 PMC8869483

[155] Liu Y , Qi X , Donnelly L , et al. Mechanisms and inhibition of porcupine‐mediated Wnt acylation. Nature. 2022;607(7920):816‐822. doi:10.1038/s41586-022-04952-2 35831507 PMC9404457

[156] Savona MR , Pollyea DA , Stock W , et al. Phase Ib study of Glasdegib, a Hedgehog pathway inhibitor, in combination with standard chemotherapy in patients with AML or high‐risk MDS. Clin Cancer Res. 2018;24(10):2294‐2303. doi:10.1158/1078-0432.CCR-17-2824 29463550

[157] Huang T , Li F , Cheng X , et al. Wnt inhibition sensitizes PD‐L1 blockade therapy by overcoming bone marrow‐derived myofibroblasts‐mediated immune resistance in tumors. Front Immunol. 2021;12:619209. doi:10.3389/fimmu.2021.619209 33790893 PMC8006364

[158] Chi SN , Yi JS , Williams PM , et al. Tazemetostat for tumors harboring SMARCB1/SMARCA4 or EZH2 alterations: results from NCI‐COG pediatric MATCH APEC1621C. J Natl Cancer Inst. 2023;115(11):1355‐1363. doi:10.1093/jnci/djad085 37228094 PMC11009504

[159] Elhanani O , Ben‐Uri R , Keren L . Spatial profiling technologies illuminate the tumor microenvironment. Cancer cell. 2023;41(3):404‐420. 36800999 10.1016/j.ccell.2023.01.010

[160] Xiao Y , Yu D . Tumor microenvironment as a therapeutic target in cancer. Pharmacol Ther. 2021;221:107753. 33259885 10.1016/j.pharmthera.2020.107753 PMC8084948

[161] Bayik D , Lathia JD . Cancer stem cell–immune cell crosstalk in tumour progression. Nat Rev Cancer. 2021;21(8):526‐536. 34103704 10.1038/s41568-021-00366-w PMC8740903

[162] Osman A , Oze M , Afify SM , et al. Tumor‐associated macrophages derived from cancer stem cells. Acta Histochem. 2020;122(8):151628. 32992123 10.1016/j.acthis.2020.151628

[163] Lo P‐K , Zhang Y , Yao Y , et al. Tumor‐associated myoepithelial cells promote the invasive progression of ductal carcinoma in situ through activation of TGFβ signaling. J Biol Chem. 2017;292(27):11466‐11484. 28512126 10.1074/jbc.M117.775080 PMC5500811

[164] Gudjonsson T , Rønnov‐Jessen L , Villadsen R , Rank F , Bissell MJ , Petersen OW . Normal and tumor‐derived myoepithelial cells differ in their ability to interact with luminal breast epithelial cells for polarity and basement membrane deposition. J Cell Sci. 2002;115(1):39‐50. 11801722 10.1242/jcs.115.1.39 PMC2933194

[165] Afify SM , Hassan G , Zahra MH , et al. Cancer stem cells as the source of tumor associated myoepithelial cells in the tumor microenvironment developing ductal carcinoma in situ. Biomaterials. 2023;301:122249. 37506511 10.1016/j.biomaterials.2023.122249 PMC10530245

[166] Coutry N , Nguyen J , Soualhi S , et al. Cross talk between Paneth and tuft cells drives dysbiosis and inflammation in the gut mucosa. Proc Natl Acad Sci. 2023;120(25):e2219431120. 37307458 10.1073/pnas.2219431120 PMC10288547

[167] Liang X , Duronio GN , Yang Y , et al. An enhancer‐driven stem cell‐like program mediated by SOX9 blocks intestinal differentiation in colorectal cancer. Gastroenterology. 2022;162(1):209‐222. 34571027 10.1053/j.gastro.2021.09.044 PMC10035046

[168] Sakahara M , Okamoto T , Srivastava U , et al. Paneth‐like cells produced from OLFM4+ stem cells support OLFM4+ stem cell growth in advanced colorectal cancer. Commun Biol. 2024;7(1):27. 38182890 10.1038/s42003-023-05504-8 PMC10770338

[169] López‐Arribillaga E , Yan B , Lobo‐Jarne T , et al. Accumulation of paneth cells in early colorectal adenomas is associated with beta‐catenin signaling and poor patient prognosis. Cells. 2021;10(11):2928. 34831152 10.3390/cells10112928 PMC8616107

[170] Shin JH , Park J , Lim J , et al. Metastasis of colon cancer requires Dickkopf‐2 to generate cancer cells with Paneth cell properties. Elife. 2024;13:RP97279. 39535280 10.7554/eLife.97279 PMC11560131

[171] van Splunder H , Villacampa P , Martínez‐Romero A , Graupera M . Pericytes in the disease spotlight. Trends Cell Biol. 2024;34(1):58‐71. 37474376 10.1016/j.tcb.2023.06.001 PMC10777571

[172] Jiang Z , Zhou J , Li L , et al. Pericytes in the tumor microenvironment. Cancer Lett. 2023;556:216074. 36682706 10.1016/j.canlet.2023.216074

[173] Guo L , Yang Q , Wei R , et al. Enhanced pericyte‐endothelial interactions through NO‐boosted extracellular vesicles drive revascularization in a mouse model of ischemic injury. Nat Commun. 2023;14(1):7334. 37957174 10.1038/s41467-023-43153-x PMC10643472

[174] Li X , Qi Q , Li Y , et al. TCAF2 in pericytes promotes colorectal cancer liver metastasis via inhibiting cold‐sensing TRPM8 channel. Adv Sci. 2023;10(30):2302717. 10.1002/advs.202302717 PMC10602580 37635201

[175] Zhou W , Chen C , Shi Y , et al. Targeting glioma stem cell‐derived pericytes disrupts the blood‐tumor barrier and improves chemotherapeutic efficacy. Cell Stem Cell. 2017;21(5):591‐603. e4. 29100012 10.1016/j.stem.2017.10.002 PMC5687837

[176] Li D , Wang L , Jiang B , Jing Y , Li X . Improving cancer immunotherapy by preventing cancer stem cell and immune cell linking in the tumor microenvironment. Biomed Pharmacother. 2024;170:116043. 38128186 10.1016/j.biopha.2023.116043

[177] Nian Z , Dou Y , Shen Y , et al. Interleukin‐34‐orchestrated tumor‐associated macrophage reprogramming is required for tumor immune escape driven by p53 inactivation. Immunity. 2024;57(10):2344‐2361. e7. 39321806 10.1016/j.immuni.2024.08.015

[178] Zhao R , Cao G , Zhang B , et al. TNF+ regulatory T cells regulate the stemness of gastric cancer cells through the IL13/STAT3 pathway. Front Oncol. 2023;13:1162938. 37534250 10.3389/fonc.2023.1162938 PMC10392945

[179] Chen X , Yang M , Yin J , et al. Tumor‐associated macrophages promote epithelial–mesenchymal transition and the cancer stem cell properties in triple‐negative breast cancer through CCL2/AKT/β‐catenin signaling. Cell Commun Signal. 2022;20(1):92. 35715860 10.1186/s12964-022-00888-2 PMC9205034

[180] Yang C‐L , Song R , Hu J‐W , et al. Integrating single‐cell and bulk RNA sequencing reveals CK19+ cancer stem cells and their specific SPP1+ tumor‐associated macrophage niche in HBV‐related hepatocellular carcinoma. Hepatol Int. 2024;18(1):73‐90. 38159218 10.1007/s12072-023-10615-9

[181] Lemaitre L , Adeniji N , Suresh A , et al. Spatial analysis reveals targetable macrophage‐mediated mechanisms of immune evasion in hepatocellular carcinoma minimal residual disease. Nat Cancer. 2024;5(10):1534‐1556. 39304772 10.1038/s43018-024-00828-8 PMC12258040

[182] Zhang Y , Song Y , Wang X , et al. An NFAT1‐C3a‐C3aR positive feedback loop in tumor‐associated macrophages promotes a glioma stem cell malignant phenotype. Cancer Immunol Res. 2024;12(3):363‐376. doi:10.1158/2326-6066.CIR-23-0418 38289255

[183] Wang L , Chao M , Han RR , et al. Single‐cell map of diverse immune phenotypes in the metastatic brain tumor microenvironment of nonsmall‐cell lung cancer. Int J Surg. 2025;111(1):1601‐1606. doi:10.1097/JS9.0000000000002088 39311908 PMC11745726

[184] Komura N , Mabuchi S , Shimura K , et al. The role of myeloid‐derived suppressor cells in increasing cancer stem‐like cells and promoting PD‐L1 expression in epithelial ovarian cancer. Cancer Immunol Immunother. 2020;69:2477‐2499. 32561967 10.1007/s00262-020-02628-2 PMC11027471

[185] Liu C , Qiang J , Deng Q , et al. ALDH1A1 activity in tumor‐initiating cells remodels myeloid‐derived suppressor cells to promote breast cancer progression. Cancer Res. 2021;81(23):5919‐5934. 34580061 10.1158/0008-5472.CAN-21-1337

[186] Otvos B , Silver DJ , Mulkearns‐Hubert EE , et al. Cancer stem cell‐secreted macrophage migration inhibitory factor stimulates myeloid derived suppressor cell function and facilitates glioblastoma immune evasion. Stem Cells. 2016;34(8):2026‐2039. doi:10.1002/stem.2393 27145382 PMC5820763

[187] Cao Y , Liu B , Cai L , et al. G9a promotes immune suppression by targeting the Fbxw7/Notch pathway in glioma stem cells. CNS Neurosci Ther. 2023;29(9):2508‐2521. 36971192 10.1111/cns.14191 PMC10401078

[188] Wang H , Zhou Q , Xie DF , Xu Q , Yang T , Wang W . LAPTM4B‐mediated hepatocellular carcinoma stem cell proliferation and MDSC migration: implications for HCC progression and sensitivity to PD‐L1 monoclonal antibody therapy. Cell Death Dis. 2024;15(2):165. 38388484 10.1038/s41419-024-06542-8 PMC10884007

[189] Zhang R , Dong M , Tu J , et al. PMN‐MDSCs modulated by CCL20 from cancer cells promoted breast cancer cell stemness through CXCL2‐CXCR2 pathway. Signal Transd Targeted Ther. 2023;8(1):97. 10.1038/s41392-023-01337-3 PMC9977784 36859354

[190] Laskowski TJ , Biederstädt A , Rezvani K . Natural killer cells in antitumour adoptive cell immunotherapy. Nat Rev Cancer. 2022;22(10):557‐575. 35879429 10.1038/s41568-022-00491-0 PMC9309992

[191] Shanley M , Daher M , Dou J , et al. Interleukin‐21 engineering enhances NK cell activity against glioblastoma via CEBPD. Cancer Cell. 2024;42(8):1450‐1466. e11. 39137729 10.1016/j.ccell.2024.07.007 PMC11370652

[192] Shaim H , Shanley M , Basar R , et al. Targeting the αv integrin/TGF‐β axis improves natural killer cell function against glioblastoma stem cells. J Clin Invest. 2021;131(14):e142116. 34138753 10.1172/JCI142116 PMC8279586

[193] Thacker G , Henry S , Nandi A , et al. Immature natural killer cells promote progression of triple‐negative breast cancer. Sci Transl Med. 2023;15(686):eabl4414. 36888695 10.1126/scitranslmed.abl4414 PMC10875969

[194] Algar S , Vázquez‐Villa H , Aguilar‐Garrido P , et al. Cancer‐stem‐cell phenotype‐guided discovery of a microbiota‐inspired synthetic compound targeting NPM1 for leukemia. JACS Au. 2024;4(5):1786‐1800. 38818079 10.1021/jacsau.3c00682 PMC11134387

[195] Tintelnot J , Xu Y , Lesker TR , et al. Microbiota‐derived 3‐IAA influences chemotherapy efficacy in pancreatic cancer. Nature. 2023;615(7950):168‐174. 36813961 10.1038/s41586-023-05728-y PMC9977685

[196] Zhu Z , Huang J , Li X , et al. Gut microbiota regulate tumor metastasis via circRNA/miRNA networks. Gut Microbes. 2020;12(1):1788891. 32686598 10.1080/19490976.2020.1788891 PMC7524358

[197] Dalmasso G , Cougnoux A , Faïs T , et al. Colibactin‐producing *Escherichia coli* enhance resistance to chemotherapeutic drugs by promoting epithelial to mesenchymal transition and cancer stem cell emergence. Gut Microbes. 2024;16(1):2310215. 38374654 10.1080/19490976.2024.2310215 PMC10880512

[198] Ma W , Zhang L , Chen W , et al. Microbiota enterotoxigenic *Bacteroides fragilis*‐secreted BFT‐1 promotes breast cancer cell stemness and chemoresistance through its functional receptor NOD1. Protein Cell. 2024;15(6):419‐440. 38437016 10.1093/procel/pwae005 PMC11131025

[199] He J , Hu W , Ouyang Q , et al. *Helicobacter pylori* infection induces stem cell‐like properties in Correa cascade of gastric cancer. Cancer Lett. 2022;542:215764. doi:10.1016/j.canlet.2022.215764 35654291

[200] Rodrigues G , Hoshino A , Kenific CM , et al. Tumour exosomal CEMIP protein promotes cancer cell colonization in brain metastasis. Nat Cell Biol. 2019;21(11):1403‐1412. doi:10.1038/s41556-019-0404-4 31685984 PMC7354005

[201] Guo R , Han D , Song X , et al. Context‐dependent regulation of Notch signaling in glial development and tumorigenesis. Sci Adv. 2023;9(45):eadi2167. doi:10.1126/sciadv.adi2167 37948517 PMC10637744

[202] Li H , Zhu J , Liu X , et al. Glioma stem cell‐derived exosomes induce the transformation of astrocytes via the miR‐3065‐5p/DLG2 signaling axis. Glia. 2024;72(5):857‐871. doi:10.1002/glia.24506 38234042

[203] Yoon SH , Lee S , Kim HS , et al. NSDHL contributes to breast cancer stem‐like cell maintenance and tumor‐initiating capacity through TGF‐beta/Smad signaling pathway in MCF‐7 tumor spheroid. BMC Cancer. 2024;24(1):1370. doi:10.1186/s12885-024-13143-3 39516821 PMC11549796

[204] Yan B , Jiang Z , Cheng L , et al. Paracrine HGF/c‐MET enhances the stem cell‐like potential and glycolysis of pancreatic cancer cells via activation of YAP/HIF‐1alpha. Exp Cell Res. 2018;371(1):63‐71. doi:10.1016/j.yexcr.2018.07.041 30056064

[205] Raj S , Kesari KK , Kumar A , et al. Molecular mechanism(s) of regulation(s) of c‐MET/HGF signaling in head and neck cancer. Mol Cancer. 2022;21(1):31. doi:10.1186/s12943-022-01503-1 35081970 PMC8790852

[206] Kroon P , Berry PA , Stower MJ , et al. JAK‐STAT blockade inhibits tumor initiation and clonogenic recovery of prostate cancer stem‐like cells. Cancer Res. 2013;73(16):5288‐5298. doi:10.1158/0008-5472.CAN-13-0874 23824741

[207] Weng YS , Tseng HY , Chen YA , et al. MCT‐1/miR‐34a/IL‐6/IL‐6R signaling axis promotes EMT progression, cancer stemness and M2 macrophage polarization in triple‐negative breast cancer. Mol Cancer. 2019;18(1):42. doi:10.1186/s12943-019-0988-0 30885232 PMC6421700

[208] Chen X , Yang M , Yin J , et al. Tumor‐associated macrophages promote epithelial‐mesenchymal transition and the cancer stem cell properties in triple‐negative breast cancer through CCL2/AKT/beta‐catenin signaling. Cell Commun Signal. 2022;20(1):92. doi:10.1186/s12964-022-00888-2 35715860 PMC9205034

[209] Anderson NR , Sheth V , Li H , et al. Microenvironmental CXCL12 deletion enhances Flt3‐ITD acute myeloid leukemia stem cell response to therapy by reducing p38 MAPK signaling. Leukemia. 2023;37(3):560‐570. doi:10.1038/s41375-022-01798-5 36550214 PMC10750268

[210] Zhan Y , Zhou Z , Zhu Z , et al. Exosome‐transmitted LUCAT1 promotes stemness transformation and chemoresistance in bladder cancer by binding to IGF2BP2. J Exp Clin Cancer Res. 2025;44(1):80. doi:10.1186/s13046-025-03330-w 40025525 PMC11874664

[211] Guo Y , Cui J , Liang X , Chen T , Lu C , Peng T . Pancreatic cancer stem cell‐derived exosomal miR‐210 mediates macrophage M2 polarization and promotes gemcitabine resistance by targeting FGFRL1. Int Immunopharmacol. 2024;127:111407. doi:10.1016/j.intimp.2023.111407 38134594

[212] Nallasamy P , Nimmakayala RK , Parte S , Are AC , Batra SK , Ponnusamy MP . Tumor microenvironment enriches the stemness features: the architectural event of therapy resistance and metastasis. Mol Cancer. 2022;21(1):225. 36550571 10.1186/s12943-022-01682-x PMC9773588

[213] Chaudhary A , Raza SS , Haque R . Transcriptional factors targeting in cancer stem cells for tumor modulation. Semin Cancer Biol. 2023;88:123‐137. 36603792 10.1016/j.semcancer.2022.12.010

[214] Shibue T , Weinberg RA . EMT, CSCs, and drug resistance: the mechanistic link and clinical implications. Nat Rev Clin Oncol. 2017;14(10):611‐629. 28397828 10.1038/nrclinonc.2017.44 PMC5720366

[215] Lee TK‐W , Guan X‐Y , Ma S . Cancer stem cells in hepatocellular carcinoma—from origin to clinical implications. Nat Rev Gastroenterol Hepatol. 2022;19(1):26‐44. 34504325 10.1038/s41575-021-00508-3

[216] Petrosyan A , Villani V , Aguiari P , et al. Identification and characterization of the Wilms tumor cancer stem cell. Adv Sci. 2023;10(20):2206787. 10.1002/advs.202206787 PMC10369255 37114795

[217] Xu C , Zhang W , Liu C . FAK downregulation suppresses stem‐like properties and migration of human colorectal cancer cells. PLoS ONE. 2023;18(4):e0284871. 37083591 10.1371/journal.pone.0284871 PMC10121060

[218] Li Z , Xu Z‐M , Chen W‐P , et al. Tumor‐repopulating cells evade ferroptosis via PCK2‐dependent phospholipid remodeling. Nat Chem Biol. 2024;20(10):1341‐1352. 38720107 10.1038/s41589-024-01612-6 PMC11427348

[219] Kellaway SG , Potluri S , Keane P , et al. Leukemic stem cells activate lineage inappropriate signalling pathways to promote their growth. Nat Commun. 2024;15(1):1359. 38355578 10.1038/s41467-024-45691-4 PMC10867020

[220] Choudhury A , Cady MA , Lucas C‐HG , et al. Perivascular NOTCH3+ stem cells drive meningioma tumorigenesis and resistance to radiotherapy. Cancer Discov. 2024;14(10):1823‐1837. 38742767 10.1158/2159-8290.CD-23-1459 PMC11452293

[221] Pang L , Dunterman M , Xuan W , et al. Circadian regulator CLOCK promotes tumor angiogenesis in glioblastoma. Cell Rep. 2023;42(2):112127. 36795563 10.1016/j.celrep.2023.112127 PMC10423747

[222] Song S , Wang L , Jiang X , et al. CircHULC accelerates the growth of human liver cancer stem cells by enhancing chromatin reprogramming and chromosomal instability via autophagy. Cell Signal. 2023;109:110772. 37321526 10.1016/j.cellsig.2023.110772

[223] Auzmendi‐Iriarte J , Matheu A . Intrinsic role of chaperone‐mediated autophagy in cancer stem cell maintenance. Autophagy. 2022;18(12):3035‐3036. 35468038 10.1080/15548627.2022.2069450 PMC9673963

[224] Sohn EJ , Kim JH , Oh S‐O , Kim J‐Y . Regulation of self‐renewal in ovarian cancer stem cells by fructose via chaperone‐mediated autophagy. Biochim Biophys Acta (BBA)‐Mol Basis of Dis. 2023;1869(6):166723. 10.1016/j.bbadis.2023.166723 37087023

[225] Yan L , Wu M , Wang T , et al. Breast cancer stem cells secrete mif to mediate tumor metabolic reprogramming that drives immune evasion. Cancer Res. 2024;84(8):1270‐1285. 38335272 10.1158/0008-5472.CAN-23-2390

[226] Wang S , Huang T , Wu Q , et al. Lactate reprograms glioblastoma immunity through CBX3‐regulated histone lactylation. J Clin Invest. 2024;134(22):e176851. 39545414 10.1172/JCI176851 PMC11563687

[227] Lv D , Gimple RC , Zhong C , et al. PDGF signaling inhibits mitophagy in glioblastoma stem cells through N6‐methyladenosine. Dev Cell. 2022;57(12):1466‐1481. e6. 35659339 10.1016/j.devcel.2022.05.007 PMC9239307

[228] Alcalá S , Sancho P , Martinelli P , et al. ISG15 and ISGylation is required for pancreatic cancer stem cell mitophagy and metabolic plasticity. Nat Commun. 2020;11(1):2682. 32472071 10.1038/s41467-020-16395-2 PMC7260233

[229] Fu L , Fan J , Maity S , McFadden G , Shi Y , Kong W . PD‐L1 interacts with Frizzled 6 to activate β‐catenin and form a positive feedback loop to promote cancer stem cell expansion. Oncogene. 2022;41(8):1100‐1113. 35034965 10.1038/s41388-021-02144-2

[230] Wei J‐R , Zhang B , Zhang Y , et al. QSOX1 facilitates dormant esophageal cancer stem cells to evade immune elimination via PD‐L1 upregulation and CD8 T cell exclusion. Proc Natl Acad Sci. 2024;121(44):e2407506121. 39432781 10.1073/pnas.2407506121 PMC11536095

[231] Li J , Xia Q , Di C , et al. Tumor cell‐intrinsic CD96 mediates chemoresistance and cancer stemness by regulating mitochondrial fatty acid β‐oxidation. Adv Sci. 2023;10(7):2202956. 10.1002/advs.202202956 PMC9982582 36581470

[232] Perkins RS , Murray G , Suthon S , et al. WNT5B drives osteosarcoma stemness, chemoresistance and metastasis. Clin Transl Med. 2024;14(5):e1670. 38689429 10.1002/ctm2.1670 PMC11061378

[233] Wang Z , Wang Q , Chen C , et al. NNMT enriches for AQP5+ cancer stem cells to drive malignant progression in early gastric cardia adenocarcinoma. Gut. 2024;73(1):63‐77. 10.1136/gutjnl-2022-328408 36977555

[234] Zhou L , Wen R , Bai C , et al. Spatial transcriptomic revealed intratumor heterogeneity and cancer stem cell enrichment in colorectal cancer metastasis. Cancer Lett. 2024;602:217181. 39159882 10.1016/j.canlet.2024.217181

[235] Peng F , Feng Y , Yu S , et al. Pan‐cancer analysis of B3GNT5 with potential implications for cancer immunotherapy and cancer stem cell stemness. PLoS ONE. 2024;19(12):e0314609. 39671359 10.1371/journal.pone.0314609 PMC11642946

[236] Zhu M , Fan H , Deng J , et al. BMI1 silencing liposomes suppress postradiotherapy cancer stemness against radioresistant hepatocellular carcinoma. ACS Nano. 2023;17(23):23405‐23421. 37988576 10.1021/acsnano.3c04636

[237] Hu Y , Zhang M , Yang T , et al. Sequential CD7 CAR T‐cell therapy and allogeneic HSCT without GVHD prophylaxis. N Engl J Med. 2024;390(16):1467‐1480. doi:10.1056/NEJMoa2313812 38657244

[238] Shi M , Wang J , Huang H , et al. Bispecific CAR T cell therapy targeting BCMA and CD19 in relapsed/refractory multiple myeloma: a phase I/II trial. Nat Commun. 2024;15(1):3371. doi:10.1038/s41467-024-47801-8 38643278 PMC11032309

[239] Kieliszek AM , Mobilio D , Upreti D , et al. Intratumoral delivery of chimeric antigen receptor T cells targeting CD133 effectively treats brain metastases. Clin Cancer Res. 2024;30(3):554‐563. doi:10.1158/1078-0432.CCR-23-1735 37787999

[240] Tan X‐Y , Li Y‐T , Li H‐H , et al. WNT2–SOX4 positive feedback loop promotes chemoresistance and tumorigenesis by inducing stem‐cell like properties in gastric cancer. Oncogene. 2023;42(41):3062‐3074. 37634009 10.1038/s41388-023-02816-1

[241] Paul S , Chatterjee S , Sinha S , et al. Veliparib (ABT‐888), a PARP inhibitor potentiates the cytotoxic activity of 5‐fluorouracil by inhibiting MMR pathway through deregulation of MSH6 in colorectal cancer stem cells. Expert Opin Ther Targets. 2023;27(10):999‐1015. 37787493 10.1080/14728222.2023.2266572

[242] Baek B‐S , Park H , Choi J‐W , Lee E‐Y , Youn J‐I , Seong S‐Y . Dendritic cells pulsed with penetratin‐OLFM4 inhibit the growth and metastasis of melanoma in mice. Biomed Pharmacother. 2024;177:117083. 38968793 10.1016/j.biopha.2024.117083

[243] Bergin CJ , Zouggar A , Mendes da Silva A , et al. The dopamine transporter antagonist vanoxerine inhibits G9a and suppresses cancer stem cell functions in colon tumors. Nat Cancer. 2024;5(3):463‐480. 38351181 10.1038/s43018-024-00727-y

[244] Beziaud L , Young CM , Alonso AM , Norkin M , Minafra AR , Huelsken J . IFNγ‐induced stem‐like state of cancer cells as a driver of metastatic progression following immunotherapy. Cell Stem Cell. 2023;30(6):818‐831. e6. 37267916 10.1016/j.stem.2023.05.007

[245] Nahas GR , Sherman LS , Sinha G , et al. Increased expression of Musashi 1 on breast cancer cells has implication to understand dormancy and survival in bone marrow. Aging (Albany NY). 2023;15(9):3230. 36996499 10.18632/aging.204620 PMC10449290

[246] Tichet M , Wullschleger S , Chryplewicz A , et al. Bispecific PD1‐IL2v and anti‐PD‐L1 break tumor immunity resistance by enhancing stem‐like tumor‐reactive CD8+ T cells and reprogramming macrophages. Immunity. 2023;56(1):162‐179. e6. 36630914 10.1016/j.immuni.2022.12.006

[247] Chakravarti M , Bera S , Dhar S , et al. Neem leaf glycoprotein disrupts exhausted CD8+ T‐cell–mediated cancer stem cell aggression. Mol Cancer Res. 2024;22(8):759‐778. 38743057 10.1158/1541-7786.MCR-23-0993

[248] Zhu P , Lu T , Chen Z , et al. 5‐Hydroxytryptamine produced by enteric serotonergic neurons initiates colorectal cancer stem cell self‐renewal and tumorigenesis. Neuron. 2022;110(14):2268‐2282. e4. 35550066 10.1016/j.neuron.2022.04.024

[249] Xie C , Liang C , Wang R , et al. Resveratrol suppresses lung cancer by targeting cancer stem‐like cells and regulating tumor microenvironment. J Nutr Biochem. 2023;112:109211. 36370924 10.1016/j.jnutbio.2022.109211

[250] Choi Y , Lee HK , Ahn D , Nam M‐W , Go R‐E , Choi K‐C . Genetically engineered neural stem cells expressing cytosine deaminase and interferon‐beta enhanced T cell‐mediated antitumor immunity against gastric cancer in a humanized mouse model. Life Sci. 2023;328:121866. 37331506 10.1016/j.lfs.2023.121866

[251] Crews LA , Ma W , Ladel L , et al. Reversal of malignant ADAR1 splice isoform switching with Rebecsinib. Cell Stem Cell. 2023;30(3):250‐263. e6. 36803553 10.1016/j.stem.2023.01.008 PMC10134781

[252] Ossami Saidy A , Peczynski C , Thieblemont C , et al. Efficacy and safety of CAR T‐cell therapy in patients with primary or secondary CNS lymphoma: a study on behalf of the EBMT and the GoCART coalition. Hemasphere. 2025;9(5):e70146. doi:10.1002/hem3.70146 40400509 PMC12093105

[253] Masoumi J , Jafarzadeh A , Abdolalizadeh J , et al. Cancer stem cell‐targeted chimeric antigen receptor (CAR)‐T cell therapy: challenges and prospects. Acta Pharm Sin B. 2021;11(7):1721‐1739. doi:10.1016/j.apsb.2020.12.015 34386318 PMC8343118

[254] Lee E , Hong JJ , Samcam Vargas G , et al. CXCR4(+) mammary gland macrophageal niche promotes tumor initiating cell activity and immune suppression during tumorigenesis. Nat Commun. 2025;16(1):4854. doi:10.1038/s41467-025-59972-z 40413176 PMC12103607

[255] Miyaguchi K , Wang H , Black KL , Shiao SL , Wang R , Yu JS . Activated T cell therapy targeting glioblastoma cancer stem cells. Sci Rep. 2023;13(1):196. 36604465 10.1038/s41598-022-27184-w PMC9814949

[256] Giron‐Michel J , Padelli M , Oberlin E , Guenou H , Duclos‐Vallee JC . State‐of‐the‐art liver cancer organoids: modeling cancer stem cell heterogeneity for personalized treatment. BioDrugs. 2025;39(2):237‐260. doi:10.1007/s40259-024-00702-0 39826071 PMC11906529

[257] Daniele S , Giacomelli C , Zappelli E , et al. Lactate dehydrogenase‐A inhibition induces human glioblastoma multiforme stem cell differentiation and death. Sci Rep. 2015;5:15556. doi:10.1038/srep15556 26494310 PMC4616042

[258] Zhang G , Zhang X , Pan W , et al. Dissecting the spatial and single‐cell transcriptomic architecture of cancer stem cell niche driving tumor progression in gastric cancer. Adv Sci (Weinh). 2025;12(18):e2413019. doi:10.1002/advs.202413019 39950944 PMC12079437

